# Visualizing the modification landscape of the human 60S ribosomal subunit at close to atomic resolution

**DOI:** 10.1093/nar/gkae1191

**Published:** 2024-12-10

**Authors:** Franziska Wiechert, Anett Unbehaun, Thiemo Sprink, Helena Seibel, Jörg Bürger, Justus Loerke, Thorsten Mielke, Christoph A Diebolder, Magdalena Schacherl, Christian M T Spahn

**Affiliations:** Institute of Medical Physics and Biophysics, Charité – Universitätsmedizin Berlin, corporate member of Freie Universität Berlin and Humboldt-Universität zu Berlin, Charitéplatz 1, 10117 Berlin, Germany; Institute of Medical Physics and Biophysics, Charité – Universitätsmedizin Berlin, corporate member of Freie Universität Berlin and Humboldt-Universität zu Berlin, Charitéplatz 1, 10117 Berlin, Germany; Core Facility for Cryo-Electron Microscopy (CFcryoEM), Charité – Universitätsmedizin Berlin, corporate member of Freie Universität Berlin and Humboldt-Universität zu Berlin, Robert-Rössle-Str. 10, 13125 Berlin, Germany; Max Delbrück Center for Molecular Medicine in the Helmholtz Association, Cryo-EM, Robert-Rössle-Str. 10, 13125 Berlin, Germany; Institute of Medical Physics and Biophysics, Charité – Universitätsmedizin Berlin, corporate member of Freie Universität Berlin and Humboldt-Universität zu Berlin, Charitéplatz 1, 10117 Berlin, Germany; Institute of Medical Physics and Biophysics, Charité – Universitätsmedizin Berlin, corporate member of Freie Universität Berlin and Humboldt-Universität zu Berlin, Charitéplatz 1, 10117 Berlin, Germany; Institute of Medical Physics and Biophysics, Charité – Universitätsmedizin Berlin, corporate member of Freie Universität Berlin and Humboldt-Universität zu Berlin, Charitéplatz 1, 10117 Berlin, Germany; Microscopy and Cryo-Electron Microscopy Service Group, Max Planck Institute for Molecular Genetics, Ihnestr. 63-73, 14195 Berlin, Germany; Core Facility for Cryo-Electron Microscopy (CFcryoEM), Charité – Universitätsmedizin Berlin, corporate member of Freie Universität Berlin and Humboldt-Universität zu Berlin, Robert-Rössle-Str. 10, 13125 Berlin, Germany; Max Delbrück Center for Molecular Medicine in the Helmholtz Association, Cryo-EM, Robert-Rössle-Str. 10, 13125 Berlin, Germany; Institute of Medical Physics and Biophysics, Charité – Universitätsmedizin Berlin, corporate member of Freie Universität Berlin and Humboldt-Universität zu Berlin, Charitéplatz 1, 10117 Berlin, Germany; Institute of Medical Physics and Biophysics, Charité – Universitätsmedizin Berlin, corporate member of Freie Universität Berlin and Humboldt-Universität zu Berlin, Charitéplatz 1, 10117 Berlin, Germany

## Abstract

Chemical modifications of ribosomal RNAs (rRNAs) and proteins expand their topological repertoire, and together with the plethora of bound ligands, fine-tune ribosomal function. Detailed knowledge of this natural composition provides important insights into ribosome genesis and function and clarifies some aspects of ribosomopathies. The discovery of new structural properties and functional aspects of ribosomes has gone hand in hand with cryo-electron microscopy (cryo-EM) and its technological development. In line with the ability to visualize atomic details – a prerequisite for identifying chemical modifications and ligands in cryo-EM maps – in this work we present the structure of the 60S ribosomal subunit from HeLa cells at the very high global resolution of 1.78 Å. We identified 113 rRNA modifications and four protein modifications including uL2-His_β-ox_216, which stabilizes the local structure near the peptidyl transferase centre via an extended hydrogen-bonding network. We can differentiate metal ions Mg^2+^ and K^+^, polyamines spermine, spermidine and putrescine and identify thousands of water molecules binding to the 60S subunit. Approaching atomic resolution cryo-EM has become a powerful tool to examine fine details of macromolecular structures that will expand our knowledge about translation and other biological processes in the future and assess the variability of the chemical space due to differences between species/tissues or varying physicochemical environment.

## Graphical Abstract

**Graphical Abstract:**
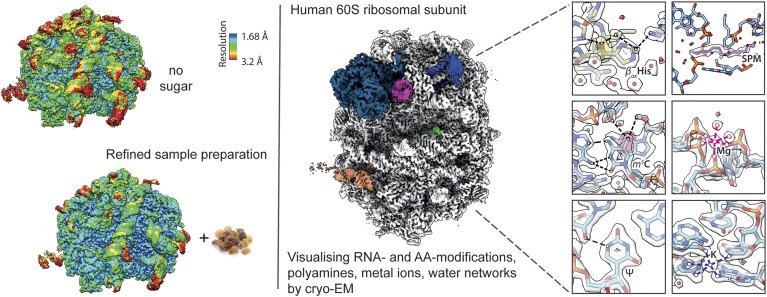

## Introduction

In each mammalian cell, up to several million 80S ribosome particles facilitate the expression of a complex and highly regulated translatome (1). The 80S ribosome is a large ribonucleoprotein (RNP) complex of 4.3 MDa that constitutes a heterodimer of a small 40S and a large 60S subunit. The human 60S subunit is built from 47 large subunit proteins and three ribosomal RNA (rRNA) chains, the 28S, 5.8S and 5S rRNAs of 5070, 157 and 121 nucleotides, respectively (2). The 28S/5.8S rRNAs folds into 101 evolutionary conserved rRNA helices assigned to six domains which are closely packed against one another in the core of the 60S, and 41 eukaryote-specific rRNA expansion segments that encase it as additional layers (3–6). The large subunit catalyses peptide bond formation, the central reaction in protein synthesis, by its peptidyl transferase centre (PTC), which is mainly created by rRNA and acts as a ribozyme. The important functional sites also include the 60S parts of the A-, P- and E-site where the three transfer RNA (tRNA)-ligands bind the ribosome by their CCA-ends, the intersubunit site for contact with the 40S subunit, the exit tunnel from which the nascent peptide progresses from the PTC to the solvent side of the ribosome, and the GTPase-associated centre to bind and to activate translational GTPases, like eEF1 and eEF2 (7,8).

An important feature of all eukaryotic ribosomes that is linked with ribosome biogenesis, rRNA folding and ribosome function is the high degree of post transcriptional modifications, which were detected in ca 3% of the nucleotides in mature mammalian rRNAs (9). rRNA modifications were explored in plants (10), yeast, protozoan parasites, drosophila (11) and mammalia (9,12), and were detected in bacterial, mitochondrial and archaeal ribosomes, although to a lesser degree than in eukaryotes (13). Universally conserved modified positions cluster in rRNA stretches of the active sites of the ribosome, including the PTC, the A-, P- and E-sites, the intersubunit sites and the peptide exit tunnel. The most common modifications in mammalian rRNA are the 2′-O-methylation at the ribose, and the conversion of uridine to pseudouridine. Other modifications of the 60S are methylations of nucleobases. While base modifications are introduced by ‘stand-alone’ methyltransferases, 2′-O-methylations and pseudouridylations are mostly conveyed by two classes of small nucleolar ribonucleoprotein (snoRNP) complexes, termed box C/D and box H/ACA snoRNPs, respectively, which via base pairing with the sno-RNA direct the catalytic protein subunit methyltransferase Nop1 (fibrillarin) (14) or pseudouridine synthetase Cbf5 (dyskerin) (15) to their specific target nucleotide. Modifications are introduced co- and post-transcriptionally by the diverse RNA-modifying enzymes during maturation of rRNA within the ribosome biogenesis pathway. It starts in the nucleoli with the transcription of the 45S and 5.8S rRNA precursors und involves processing, modification and folding of rRNAs which together with ribosomal proteins and biogenesis factors assemble into pre-ribosome particles that are exported from the nucleoplasm into the cytoplasm to maturate into the 40S and 60S ribosomal subunits which can start protein synthesis (16).

In general, modification of the rRNA nucleotides expand the interaction repertoire of the nucleoside and thus stabilize the secondary and tertiary structure of the rRNA and support rRNA folding and maturation (13). In mature ribosomes, rRNA modifications affect the strength of the intersubunit contacts, the conformational dynamics of the ribosome and the interaction with the tRNA and protein ligands (17). rRNA modifications were shown to affect the speed and the fidelity of protein synthesis, and thus participate in translation regulation (18). Global inhibition of pseudouridinylation or 2′O-methylation caused specific defects in translation of messenger RNAs (mRNAs) with internal ribosomal entry sites (IRES) or regulatory RNA elements, and genetic experiments in human cells and animal models could show, that site specific 2′O-Met modifications in the 18S and 28S rRNAs determine the expression of mRNAs which determine cell division and differentiation (19,20). Interestingly, there is polymorphism regarding the occupancy of rRNA modifications at non-conserved eukaryote specific modification sites. In human cells, almost 50% of all modifications occur at sub-stochiometric levels, and modification patterns can change under certain conditions, for instance, during development and cell differentiation (9,21). In addition, some modifications of ribosomal proteins are also known, for instance the post-translational oxidation of His and methylation of Lys, and His could be linked with translational control or stabilization of ribosomal structure (22,23). Heterogeneity in rRNA modifications is in line with the emerging concept of specific ribosomes, according to which subpopulations of ribosomes with variations in their core constitution translate specific mRNA subsets (13,24,25). Several examples of a physiological role of ribosomes with alterations in the stoichiometry of ribosomal proteins or the expression of paralogues in translational control have been described in recent years. Ribosome heterogeneity is linked with human diseases, for instance, ribosomopathies, and ribosomes with altered occupancy of rRNA modifications were reported to be involved in cancer development or were detected in cancer tissues (26–29).

Understanding the role of rRNA modifications in biology and pathology requires their comprehensive and quantitative mapping. The detection of rRNA modifications was initially conducted by thin-layer chromatography of hydrolysed rRNA (30). Later, quantitative mass spectrometry of digested rRNA revealed a complete rRNA modification pattern and quantitation of the modification occupancy of human 80S ribosomes (9). Now, several methods of rRNA analysis involving high throughput sequencing are available, among them a specific method for quantitative mapping of pseudouridinylations involving nucleotide derivatization, and direct nanopore sequencing (31,32). These methods allow fast and quantitative analysis of a biological samples or small starting material of clinical isolates. However, direct visualization of rRNA modifications in a high-resolution structure will provide information on their interaction pattern and thus, their structural role.

Protein synthesis critically requires several small cofactor molecules, mainly monovalent potassium (K^+^) and divalent magnesium (Mg^2+^) ions, and polyamines, which are small aliphatic polycations mainly generated from the amino acid ornithine by specific biosynthesis pathways (33). All of these cofactors affect several components of translation, for instance the folding and activity of tRNA and mRNA ligands and the structure and activity of the ribosomal GTPases. Both, the structure and the function of the ribosome itself, and the stable association of ribosomal subunits depend on the presence of these molecules *in vitro* (34–38); and their homeostasis also affects ribosome biogenesis and function *in vivo*. In general, the contribution of ions and polyamines on ribosome structure could be explained by the folding of rRNA helices into domains and the final compact monolithic structure of the large subunit. The long rRNA chain with its negative phosphates causes repulsion which is overcome by counter ion condensation and specific metal-induced RNA folds (39). In line with this, Mg^2+^ and polyamines abundantly associate with the ribosome fraction and polyamine synthesis is crucial for biogenesis of the 60S, but not the 40S subunit (33,34,37,40,41). According to biochemical data, polyamines are positioned in prominent functional sites of the large subunit (41) and lower the optimal Mg^2+^-concentration required for functional activity of the ribosome in *in vitro* reactions (40), which is based on some overlap of polyamine and metal binding sites as described in crosslinking studies in bacterial ribosomes (42) all of which were positioned in prominent functional sites of the large subunit (41) Although polyamines participate in translation in all organisms, spermidine (SPD) and spermine (SPM) are the dominating in eukaryotes, and putrescine (PUT) in *Escherichia coli* (43).

Cryo-electron microscopy (cryo-EM) has become the preferred method for structural studies of the ribosome (44) and enabled simultaneous investigation of structural and functional details within one sample (45). Due to technical and computational developments, and optimized sample preparations in recent years, cryo-EM has even reached atomic resolution in the analysis of macromolecular structures. Thus, such achievements need to be made amenable for the ribosome, in order to obtain the atomic details of the rRNA and protein building blocks and their modifications, solvent sphere and small molecule ligand composition.

In this work we present the structure of the HeLa 60S ribosomal subunit at 1.78 Å global resolution, with local resolution ranging down to the Nyquist limit of 1.232 Å, that reveals the above-mentioned details at atomic level. The quality of the map allows us to unambiguously identify protein and RNA modifications and to build an accurate atomic model to identify interactions between RNAs, proteins as well as the surrounding solvent molecules. Water molecules, metal ions and polyamines provide extended hydrogen-bond networks that stabilize the structure locally, while also allowing a high degree of structural flexibility. Our study shows that cryo-EM is a suitable method to visualize interactions at atomic level, which in the future will allow us to expand our knowledge about translation and other molecular and biological processes.

## Materials and methods

### Purification of HeLa 60S subunits

HeLa 60S subunits were isolated from cytoplasmic extracts of HeLa cells, involving puromycin treatment and sucrose gradient centrifugation in high salt, adopted from published protocols (46–48). Standard precautions regarding laboratory equipment and solutions were taken to prevent nucleolytic degradation of ribosomes, and all preparative steps and centrifugations were performed fast and in the cold. One tube of frozen HeLa cells was thawed in ice water and was mixed with two volumes of hypotonic buffer A [20 mM 4-(2-hydroxyethyl)-1-piperazineethanesulfonic acid (HEPES), pH 7.5, 10 mM KCl, 1.5 mM MgCl_2_, 25 μM hemin, 2 mM dithiothreitol (DTT), 5 μM MG-132 and 1× complete ethylenediaminetetraacetic acid (EDTA)-free protease inhibitor]. Cells were swollen on ice for 30 min and were broken in a glass Dounce homogenizer by 30 manual strokes. The postnuclear and postmitochondrial fraction (PMF) of the cell homogenate were obtained by stepwise centrifugation in a JA-30.50Ti rotor at 1800 rpm for 5 min and at 8500 rpm for 15 min, respectively. One volume of the PMF was then mixed with one-third of buffer B (20 mM HEPES, pH 7.5, 100 mM KCl, 2.5 mM MgCl_2_, 1 M sucrose, 2 mM DTT) and a crude ribosomal pellet was obtained by centrifugation for 4 or 5 h at 45 000 rpm in a Ti50.2 rotor. The pellet was thawed on ice and was dissolved in buffer C (20 mM HEPES, pH 7.5, 50 mM KCl, 4 mM MgCl_2_, 0.1 mM EDTA, 250 mM sucrose, 2 U/ml RNAse inhibitor, 2 mM DTT) by gentle stroking with a glass stick, followed by magnetic stirring in an ice water bath. The salt concentration was increased to 500 mM KCl, by dropwise addition of 4 M KCl, and a pellet of salt-washed crude ribosomes was obtained by ultracentrifugation and was resuspended in buffer C, as described above. The ribosome resuspension was treated with 0.5% Igepal for 10 min on a rotating wheel to release ribosomes from membranes, followed by incubation with 1 mM puromycin for 20 min on ice, and for 10 min at 37°C. The salt concentration was increased to 0.5 M KCl by addition of 4 M KCl, and the resuspension was loaded on 10%–30% sucrose gradients in buffer D (20 mM HEPES, pH 7.5, 500 mM KCl, 4 mM MgCl_2_, 2 mM DTT) and subjected to ultracentrifugation at 20 600 rpm for 20 h at 4°C on an SW32 rotor (Beckman). The gradients were manually fractionated, and separated peak fractions of 60S subunits were collected. To avoid a partial loss of their functional activity by pelleting subunits (48), we then performed buffer exchange by gel filtration, or dilution and ultrafiltration.

For buffer exchange, the sample was subjected to size exclusion chromatography on Superose 6 columns in buffer E (20 mM HEPES, pH 7.5, 2 mM MgCl_2_, 90 mM KCl, 0.5 mM SPD, 0.04 mM SPM, 2 mM DTT) or was diluted 6× in buffer F (20 mM HEPES, pH 7.5, 10 mM KCl, 2 mM MgCl_2,_ 2 mM DTT) or buffer F* (buffer F supplemented with 8.56% sucrose) to adjust the sucrose in the storage buffer to 0%, 4% or 12%, respectively. Finally, the 60S subunit suspension was concentrated on Amicon Ultracel filter units with a 10 kDa cutoff was snap frozen in 10 μl aliquots and stored at −80°C.

### Cryo-EM sample preparation, data collection and refinement

Concentrated HeLa 60S ribosomal subunits were diluted to 200 nM in buffer G (20 mM HEPES, pH 7.5, 100 mM KCl, 1.5 mM MgCl_2_, 0.5 mM SPD, 0.04 mM SPM, 1 mM DTT) and 4 μl were spotted onto glow-discharged holey carbon grids coated with a continuous thin carbon film (Quantifoil Cu200 R2/2+) and incubated for 40 s at 4°C, 85% relative humidity (RH). The excess of liquid was blotted away for 2 s and the grids were plunge frozen using the Vitrobot Mark IV. Data was acquired using a Titan Krios G3i microscope at 300 kV with a K3 direct electron detector at a nominal magnification of 105 kx with a pixel size of 0.412 Å in super-resolution mode, with a dose of 1 e^−^/Å^2^ per fraction totalling to 49 e^−^/Å^2^. In total 16 983 micrographs from one grid were collected with defocus values ranging from −0.5 to −2 μm. Data collection and refinement statistics are summarized in Supplementary Table S1.

### Single-particle analysis and refinement

Initial motion correction was done using Warp v1.0.9 (49), followed by contrast transfer function (CTF) estimation using GCTF v1.06 (50) and particle picking in Gautomatch v0.56 (developed by Kai Zhang) using a blob picker with a diameter of 250 Å. About 1.37 million particle images were extracted in RELION v3. (51) 1 with a box size of 960 px (decimated to 160 px; 2.472 Å/px) and imported to CryoSPARC v3.3.1 (52). After *ab initio* reconstruction the particles underwent hetero-refinements to remove non-ribosomal particles and 80S ribosomal particles. About 880k 60S ribosomal subunit particles were re-extracted at a pixel size of 0.832 Å and underwent non-uniform-refinement (correcting for higher order aberrations), CTF per particle refinement and 3D-reconstruction with Ewald’s sphere correction to a resolution of 1.93 Å. The refined particles were exported to RELION v3.1 for Bayesian polishing. Polished particles with a pixel size of 0.616 Å were re-imported to CryoSPARC and underwent a final non-uniform-refinement (correcting for higher order aberrations) and reconstruction with Ewald's sphere correction, which improved the map to a final global resolution of 1.78 Å (FSC_0.143_). Processing of undecimated data with a pixel size of 0.412 did not improve this map any further (data not shown). Single particle data processing scheme is provided in Supplementary Figure S6.

To identify distinct conformations of flexible segments, three rounds of 3D-variability analysis based on principal component analysis (PCA) were done in CryoSPARC with focus masks covering one expansion segment (ES) at a time. For further analysis of ES39 two additional rounds of 3D-variability analysis were performed. Alternatively, 3D classification based on PCA with 30 classes was done in CryoSPARC.

### Atomic model building

Interactive model building based on PDB-ID: 6eko (12) was performed in C*oot* v0.9.6.4 (53) using an auto sharpened map (CryoSPARC) and additionally a low-pass-filtered map to 4 Å for the more mobile regions of the 60S structure. Each residue of the model was checked individually for modifications and adjusted manually with a subsequent refinement in Phenix v.1.19 using phenix.refine (54,55). Polyamines and metals were added while searching for unmodelled blobs in C*oot*. Water molecules were added using phenix.douse (54) and visually validated in C*oot*. The model was validated using Molprobity (56).

### Cryo-electron tomographic (cryo-ET) data collection

Tilt series data collection was performed on a Titan Krios G3i microscope equipped with a K3 direct electron detector using SerialEM version 4.1beta (57), employing the dose-symmetric scheme (58) and the PACE-tomo script (59). The following settings were used: magnification 19 500×, pixel size on sample 2.2425 Å, tilt range −48° to + 48° with 3° interval, K3 camera in super-resolution mode, target dose rate on camera 7.8 to 8.4 e^−^/pixel/s, 10 frames per tilt image, constant exposure time for each tilt and total dose 102–109 e^−^/Å^2^. For different tilt series, the target defocus values ranged from −3 μm to −7 μm. In total, 70 tilt-series for 60S_0.2S_ grid preparation and 31 tilt-series for 60S_1.2S_ grid preparation were used for data processing and analysis. Tilt data acquisition and processing statistics are summarized in Supplementary Table S2.

### Image processing and structure refinement

Cryo-ET data was processed in Warp v1.0.9 (49), including frame motion correction, CTF estimation and tilt series sorting. Tilt series alignment was done in AreTomo (60). Tomograms were first reconstructed at 12 Å voxel size in Warp, after importing the AreTomo alignments. Then 60S ribosomal subunit picking was done using template matching in Warp and the top 3000 (for 60S_0.2S_) or 4000 (for 60S_1.2S_) ranking cross correlation hits for every tomogram were extracted based on the value for the picking figure of merit (_rlnAutopickFigureOfMerit). In total, 210 000 subtomograms for 60S_0.2S_ grid preparations and 124 000 subtomograms for 60S_1.2S_ grid preparations were extracted at bin 4 (voxel size 8.97 Å) in Warp and subjected to 3D classification to remove false positives in RELION v4.0 (61). Subsequently, the cleaned subtomograms were mapped back into the tomograms with the ArtiaX plugin (62) in ChimeraX v1.7 (63) for visualization. The relative ice thickness of tomograms (Supplementary Table S3) was calculated based on three thickness measurements per tomogram (within each hole) using 3dmod (64).

#### Reagents and major instrumentation

SPD trihydrochloride (Sigma-Aldrich Chemie GmbH, Taufkirchen, Germany, #85578), SPM (Sigma-Aldrich Chemie GmbH, Taufkirchen, Germany, #85605), Ti50.2 rotor (Beckman, Krefeld, Germany), Superose 6 column (Cytiva, Freiburg im Breisgau, Germany), Ti50.2 rotor (Beckman, Krefeld, Germany), Amicon Ultracel filter unit with a 10 kDa cutoff (Merck Millipore, Darmstadt, Germany), Holey carbon grids coated with a continuous thin carbon film Cu200 R2/2+ (Quantifoil, Großlöbichau, Germany), Vitrobot Mark IV (Thermo Fischer Scientific, Eindhoven, The Netherlands), Titan Krios G3i microscope (Thermo Fischer Scientific, Eindhoven, The Netherlands), K3 direct electron detector (Gatan, Pleasanton, USA).

#### Biological Resources

Frozen HeLa cell pellets (Ipracell, Mons, Belgium, # CC-01–10-50, https://www.ipracell.be/product/hela-pellet/).

#### Data availability and used software

Cryo-EM map: Electron Microscopy Data Bank (EMDB) under accession code EMD-18765. Atomic model: Protein Data Bank (PDB) under accession code 8QYX. Warp v1.0.9 – frame motion correction, CTF estimation, tilt sorting (49), GCTF v1.06 – CTF-correction (50), Gautomatch v0.56 – particle picking (developed by Kai Zhang) RELION v3.1 – particle extraction, Bayesian polishing (51), CryoSPARC v3.3.1 – 2D classification, 3D *ab initio* reconstruction, hetero refinement, 3D refinement, higher-order-aberration correction, Ewald’s sphere correction, map sharpening (52), C*oot* v0.9.6.4 – interactive model building (53), Phenix v.1.19 – 3D-structure refinement, automated identification of water molecules (54), MolProbity – structure evaluation (56), SerialEM version 4.1beta (57) – cryo-ET data acquisition, PACE-tomo script (59) – beam shift/image shift-based multiple tilt-series acquisition, AreTomo - automated marker-free cryo-electron tomographic alignment (60), RELION v4.0 – 3D classification of subtomograms (61), ChimeraX v1.7 – structure visualization (63), ArtiaX plugin – tomogram and subtomogram visualization (62), 3dmod – determination of ice thickness (65), BLAST – sequence search and alignment (66).

## Results and discussion

### Approaching atomic resolution

To prepare a specimen suited for high-resolution cryo-EM analysis we prepared ribosomal 60S subunits by classical sucrose gradient centrifugation. Because sucrose interferes with the contrast of the particles in cryo-EM micrographs (67), we initially had performed gel filtration to adjust the buffer to minimal sucrose concentrations. We noticed, however, on micrographs that these samples prepared without the addition of sucrose exhibited evidence of ribosome aggregation and denaturation (Supplementary Figure S1). Therefore, we considered keeping some sucrose in the final sample (0.2% sucrose for 200 nM 60S, subscripted ‘0.2S’; Supplementary Figure S1). Such sample conditions mediated a more homogenous distribution of the particles on the thin carbon over the grid holes, compared to no sucrose (Supplementary Figure S2), and enabled us to acquire microscopic data, which was reconstructed to 2.04 Å (FSC_0.143_) global resolution (Supplementary Figure S3).

**Figure 1.**
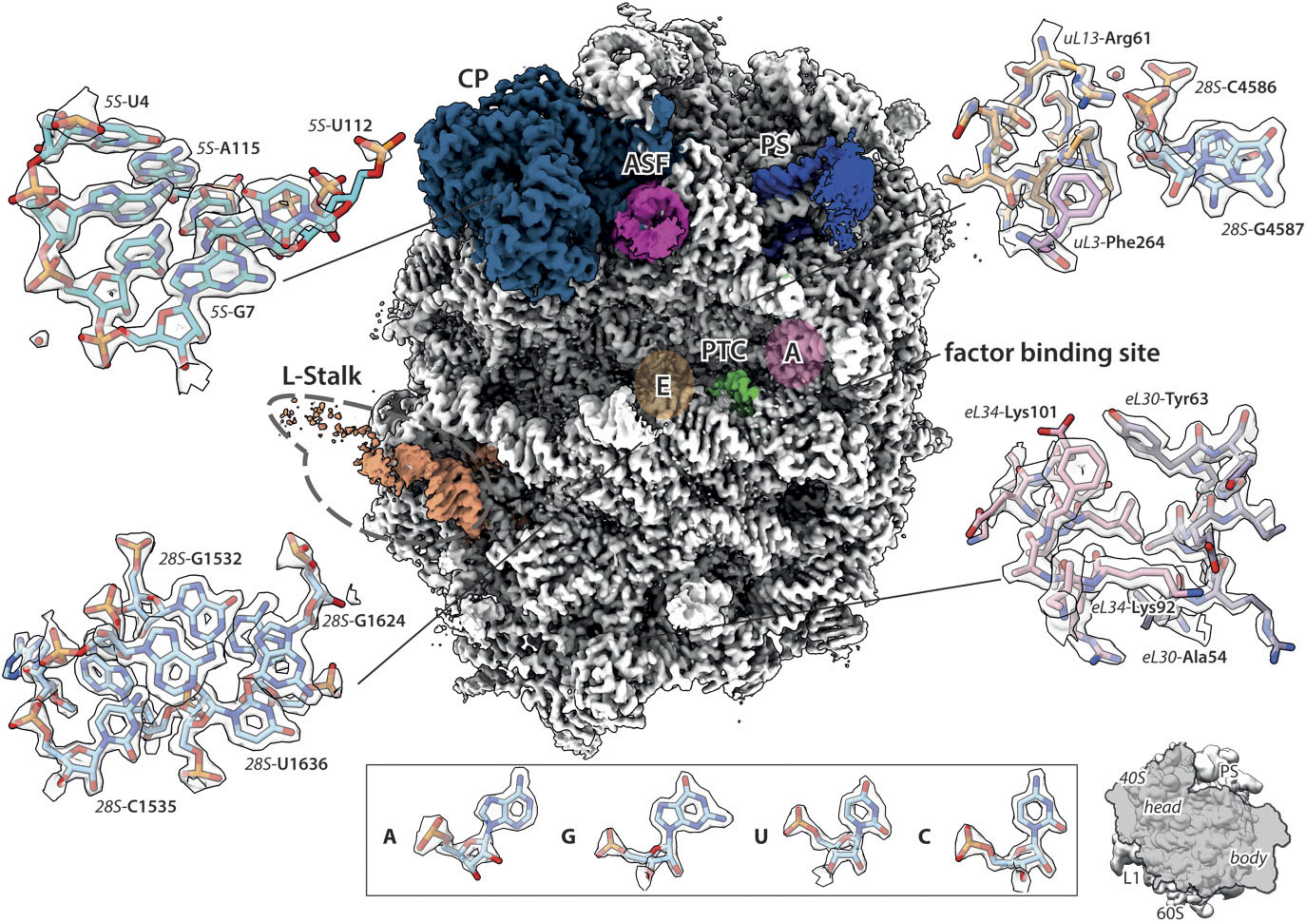
Cryo-EM reconstruction of the human ribosomal 60S subunit derived from HeLa-cells. Refined map of the 60S ribosomal subunit coloured according to its structural features: Central protuberance (CP, cyan), P-stalk (PS, blue), A-site finger (ASF, pink) and PTC (green). The A-site (A, light pink) and E-site (E, brown) are also indicated as well as the L-stalk (orange) and the factor binding site. Close-ups show representative randomly picked high-resolution features of the map with the corresponding model, including rRNA and polypeptide. The inset shows exemplary densities and models for all four standard ribonucleotides. The interface of the 40S subunit in respect to the 60S subunit in indicated in a miniature on the lower right bottom.

However, we realized that the 60S_0.2S_ cryo-EM map exhibited some disorder at the periphery (Supplementary Figure S2), mainly at rRNA helices H25ES7a and H25ES7e (expansion segments 7a and 7e), H30ES9 (expansion segment 9), H42-44 (GTPase-associated centre), H54ES20 (expansion segment 20) and H77-78 (L1 stalk) and H98 (ES39). This could point to a defined interface that 60S_0.2S_ presents either to the 2 nm carbon layer of the grid or, more likely, to the air–water interface and which imposes disorder at this very hydrophobic surface (68). Exploring, if additional sucrose may overcome this, we increased sucrose to final 1.2% (Supplementary Figure S1). Subsequent dilution to 1.2% sucrose (for 200 nM 60S, subscripted ‘1.2S’) for the final cryo-EM sample preparation, enabled us to obtain the here presented high-quality cryo-EM map at 1.78 Å (FSC_0.143_) global resolution (Figure 1 and Supplementary Figure S3).

Compared to 60S_0.2S_, the 60S_1.2S_ 3D reconstruction yielded a density map less disordered at the periphery of the subunit (Supplementary Figure S2) and revealed great molecular detail including thousands of defined water molecules and ions as well as polyamines, modified amino acids and ribonucleotides (Figure 1). We acquired 70 tilt series for a representative grid preparation of 60S_0.2S_ and 31 tilt series for 60S_1.2S_, reconstructed tomograms and subtomograms (Supplementary Figure S4) to determine the location of particles in the vitreous ice layer (Supplementary Figure S5) and to approximate the ice layer thickness (Supplementary Table S3). For 60S_0.2S_ subunits the average ice thickness of 53.4 ± 6.6 nm within the tomograms was measured (median across 12 representative tomograms, values range from 43 nm to 122 nm depending on the position over the hole). Their distribution within the ice above the carbon holes is inhomogeneous, with a considerable extent of particle aggregation at the hole edges (Supplementary Figure S5A and B). By contrast, ice thickness shortened to 49.3 ± 3.0 nm (median across 12 representative tomograms, values range from 37 nm to 56 nm) for 60S_1.2S_, accompanied by a more compact and homogeneous particle layer and less particle aggregation visible in the tomograms (Supplementary Figure S5C and D). The higher sucrose concentration clearly leads to more ‘single’ 60S subunits within the vitreous ice layer, also indicated by the absence of 3D classes showing a close ‘neighbour’ molecule for 60S_1.2S_ in the final 3D classification (Supplementary Figure S4B), in contrast to 60S_0.2S_, for which 11.7% of all particles exhibit this behaviour (Supplementary Figure S4A, classes c1 and c4).

Interestingly, the sugar-dependent behaviour during grid preparation is limited to isolated ribosomal subunits. Polysomes and fully assembled 80S ribosomes prepared by sucrose density gradients do not require preservation of sucrose and can be diluted to any ‘ribosome buffer’ without deteriorating effects during grid preparation (45). Possibly, isolated ribosomal subunits align to the carbon-water and air–water boundaries along different protein/RNA interfaces than 80S ribosomes and polysomes do. We consider that the sucrose homogeneously pervades almost the whole volume of the applied sample. However, sucrose is known to be repelled by the air–water interface and to be absent from the depletion layer, which is typically slightly thinner than 1.0 nm, closest to the air interface (69). This effect could cause the ribosomal subunits to also be repelled from the air–water interface, which would protect them from denaturation. In addition, the water evaporation rate decreases in sugar solutions, which may produce more uniform water film prior to plunge freezing (67). The higher resolution achieved for the 60S_1.2S_ sample could alternatively be explained by a protective role of sucrose during the concentration step on the filtration membrane. The presence of sucrose stabilizes protein structures by increasing the apparent activation energy of the unfolding reaction (70). The effect is most probably conferred on the protein through the increase in the solvent cohesive force when sucrose is added to water in the solvent system. Therefore, sucrose may be considered as a general protectant for macromolecules during vitrification.

Recently, efforts were made by other research groups to improve sample preparation to obtain high-resolution reconstructions of 60S and 40S ribosomal subunits (71,72). There, thin carbon-coated grids were functionalized with PMA (1-pyrenemethylamine), which generates a positively charged grid surface. By that means, 60S and 40S ribosomal particles may orient differently based on the interaction of their negatively charged RNA phosphate backbone than when absorbed on thin negatively charged carbon, glow-discharged by air plasma (73), as performed in our study.

### High-resolution map of human 60S

**Figure 2.**
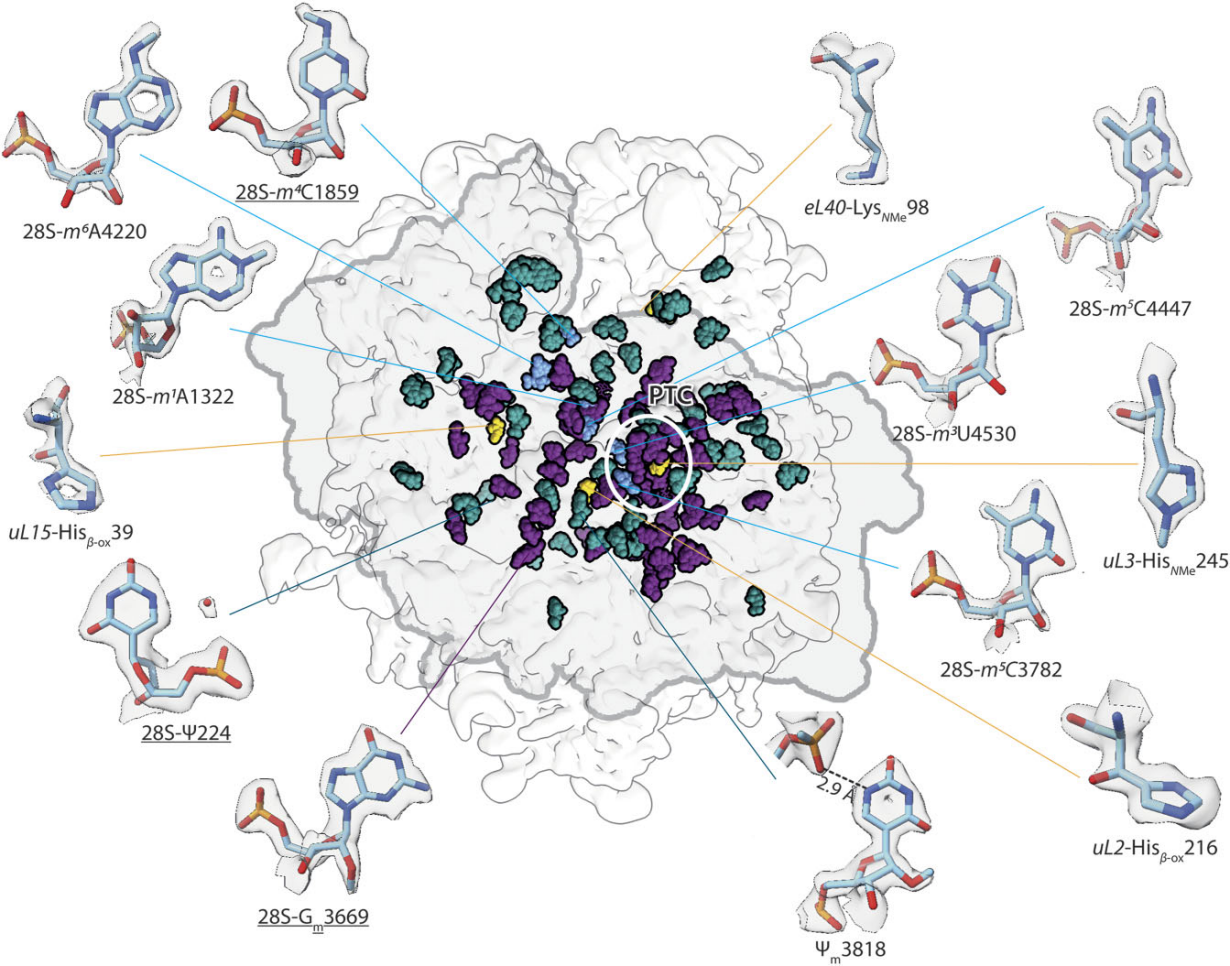
Localization of all identified rRNA- and protein modifications in the human 60S ribosomal subunit. All identified modifications were mapped onto the 60S ribosomal subunit map (depicted as transparent map in the centre), highlighted as spheres and coloured according to their identity, namely: pseudouridinylations (teal) and ribose methylations (purple), as well as more rare nucleobase modifications (light blue) and amino acid modifications in ribosomal proteins (yellow). Modifications cluster in the centre of the 60S ribosomal subunit, close the PTC (white circle) and near the interface to the 40S ribosomal subunit (indicated with grey outline). Close-ups, aligned around the map, show selected rare modifications as sticks surrounded by transparent density shown at a contour level of 8σ. Underlined modifications are those for which indications were found here for the first time.

**Figure 3.**
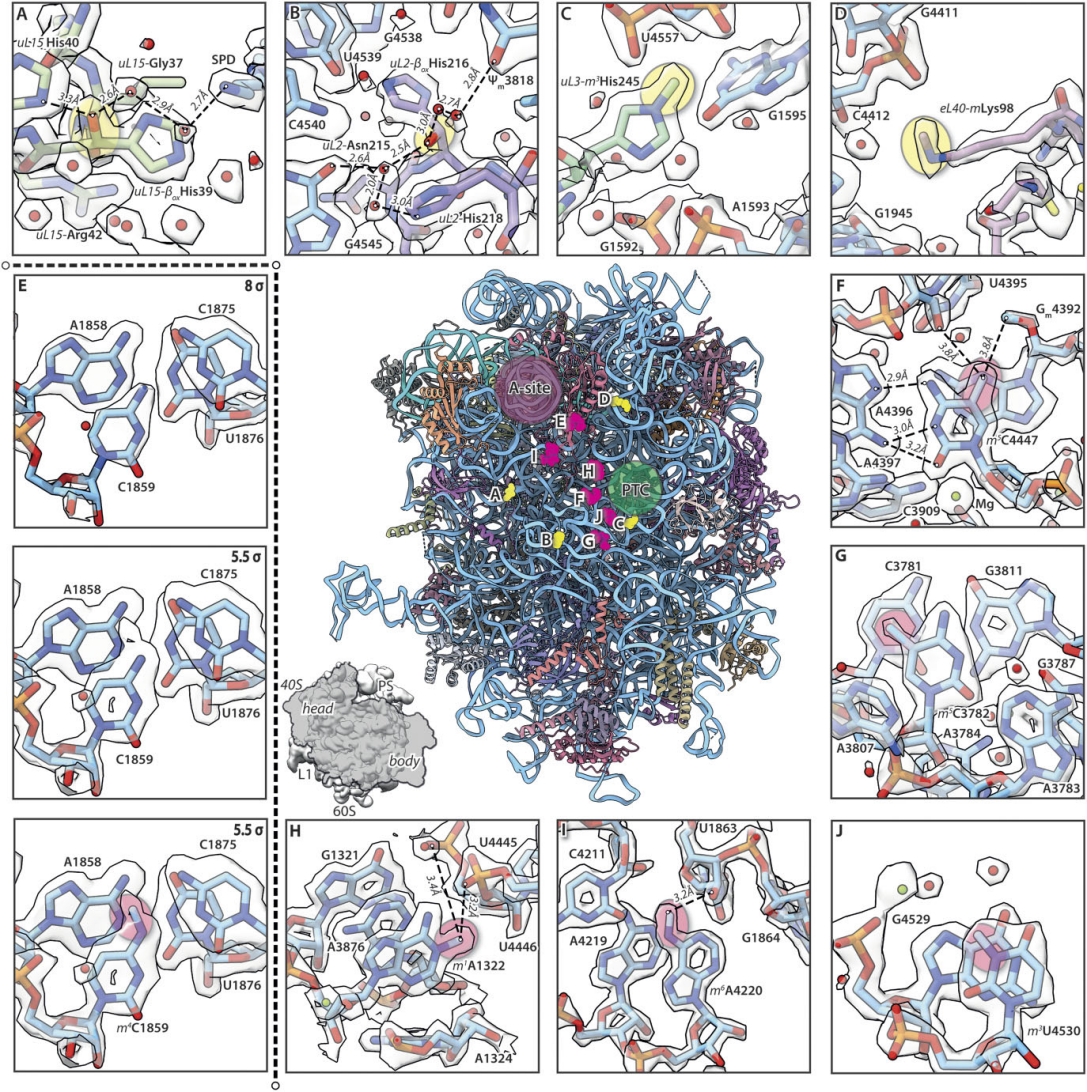
Overview of protein and nucleobase modifications in the human 60S ribosomal subunit. Identified protein modifications were β-oxygenation of uL15 His39 (**A**) and uL2 His216 (**B**) as well as N-methylation of uL3 His245 (**C**) and of eL40 Lys98 (**D**). The three histidine modifications (A–C) are close to the PTC while eL40 mLys98 is found at the surface of the 60S ribosomal subunit [panel (D)]. (**E**)> Nucleobase modifications that can be identified visually. From top to bottom: Map at contour level of 8σ does not display the modification, but at 5.5σ level clear additional density is visible for ribonucleotide C1859, which can be interpreted as N4-methylcytidine (red circle). Five different types of nucleobase methylations were found. These were (**E**)> N4-methylcytidine (m^4^C), (**F, G**) 5-methylcytidine (m^5^C), (**H**) N1-methyladenosine (m^1^A), **(I)** N6-methyladenosine (m^6^A) and **(J)** 3-methyluridine (m^3^U), located close to the peptidyl transferase centre (encircled) and at the interface to the 40S ribosomal subunit, respectively, indicated as small inset. The methylations provide more stability by enhancing base-stacking [panels (E), (F) and (J)] or by allowing further interactions to neighbouring nucleotides [panels (G–I)]. Cartoon representation of the human 60S ribosomal subunit in the centre, surrounded by examples of modified amino acids [panels (A–D), highlighted with yellow circle] and nucleobases [panels (E–J), red circle] shown as sticks with their transparent density at a contour level of 5.5σ. Selected interactions are indicated as dashed lines with distances in Å. Water molecules (red spheres) and magnesium ions (green spheres) are also indicated.

**Figure 4.**
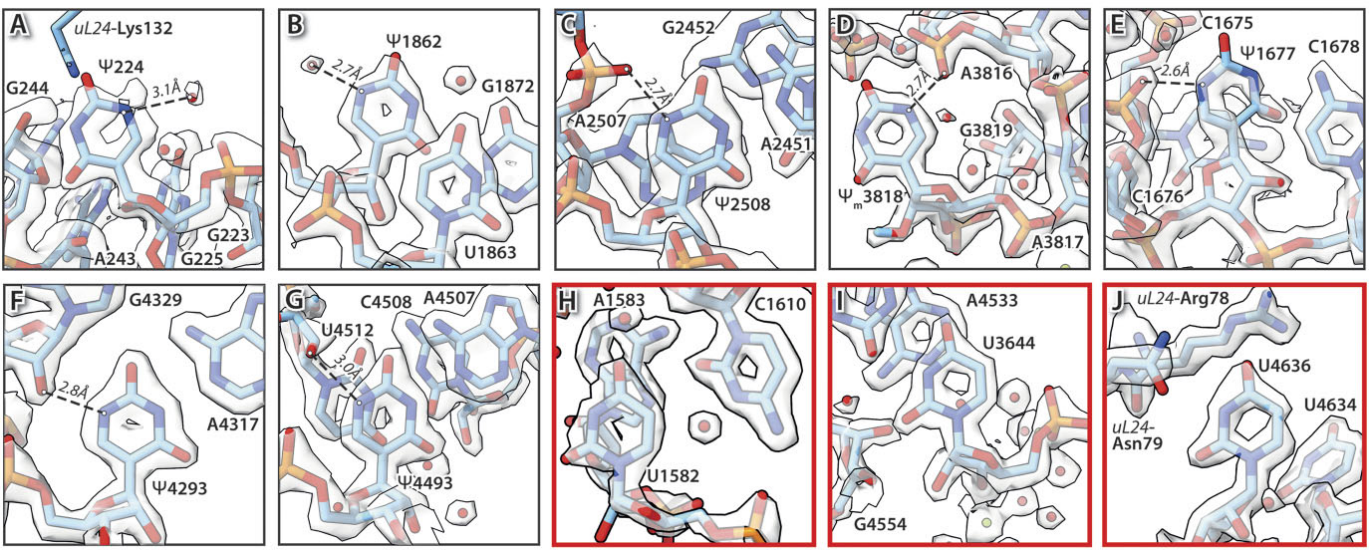
Pseudouridinylations found in the human 60S ribosomal subunit. Pseudouridines in electron density maps were identified by their binding pattern. The electron donor can be either a water molecule **(A, B)**, the phosphate **(C–E)** or the ribose 2′-hydroxide **(F, G)** of a neighbouring nucleotide, forming a hydrogen bond to atom N1 of pseudouridine. In contrast uridines **(H–J)** lack these additional and stabilizing interactions. Nucleotides shown as sticks with their transparent density at a contour level of 5.0σ for panels (A) and (E); and 5.5σ for all other panels. Selected interactions are indicated as dashed lines with distances in Å. Water molecules (red spheres) are also indicated, where present.

**Figure 5.**
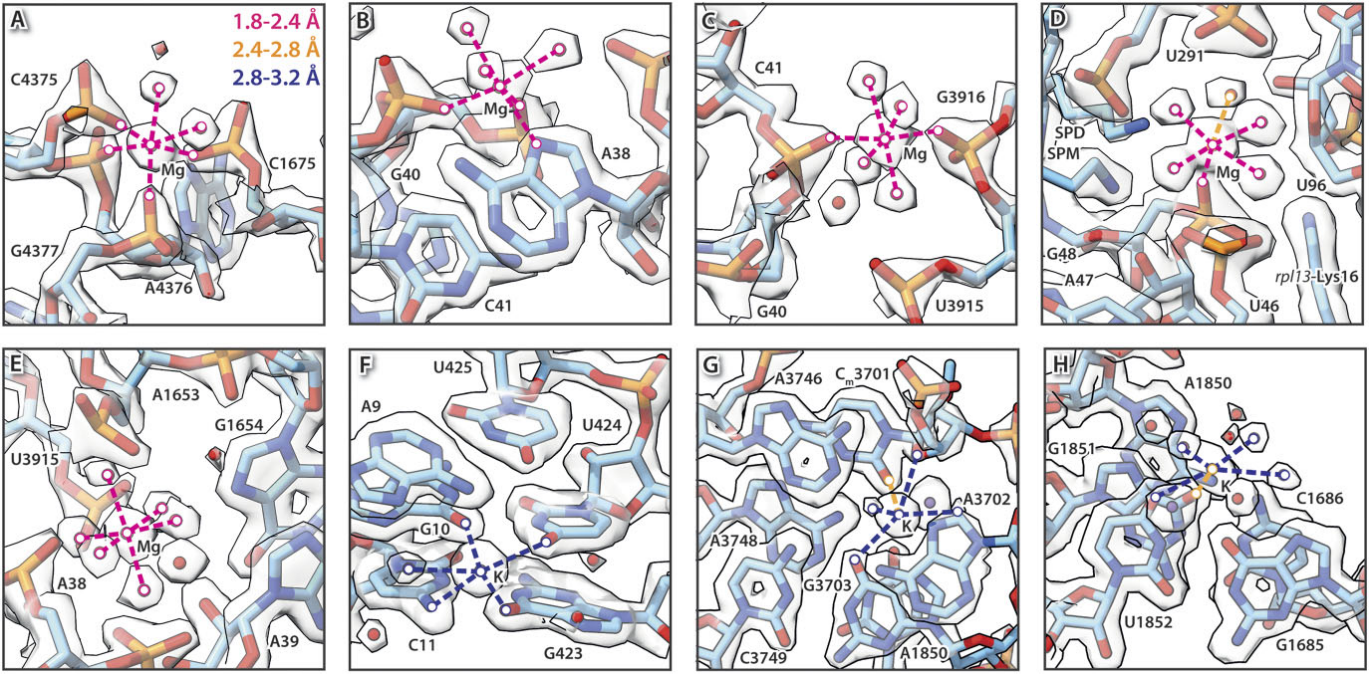
Ion coordination sites in the HeLa 60S ribosomal subunit. (**A**–**E**) In the HeLa 60S ribosomal subunits various coordination patterns can be observed, ranging from coordinating to (A) four phosphates and two waters to (E) being surrounded by six water molecules. In contrast, potassium (K) prefers a pentagonal coordination and interacts with nucleobases and water molecules (**F,G**). K is shown as purple sphere and Mg as a green sphere. The coordination bond distances are indicated, and colour coded, as shown in (A).

**Figure 6.**
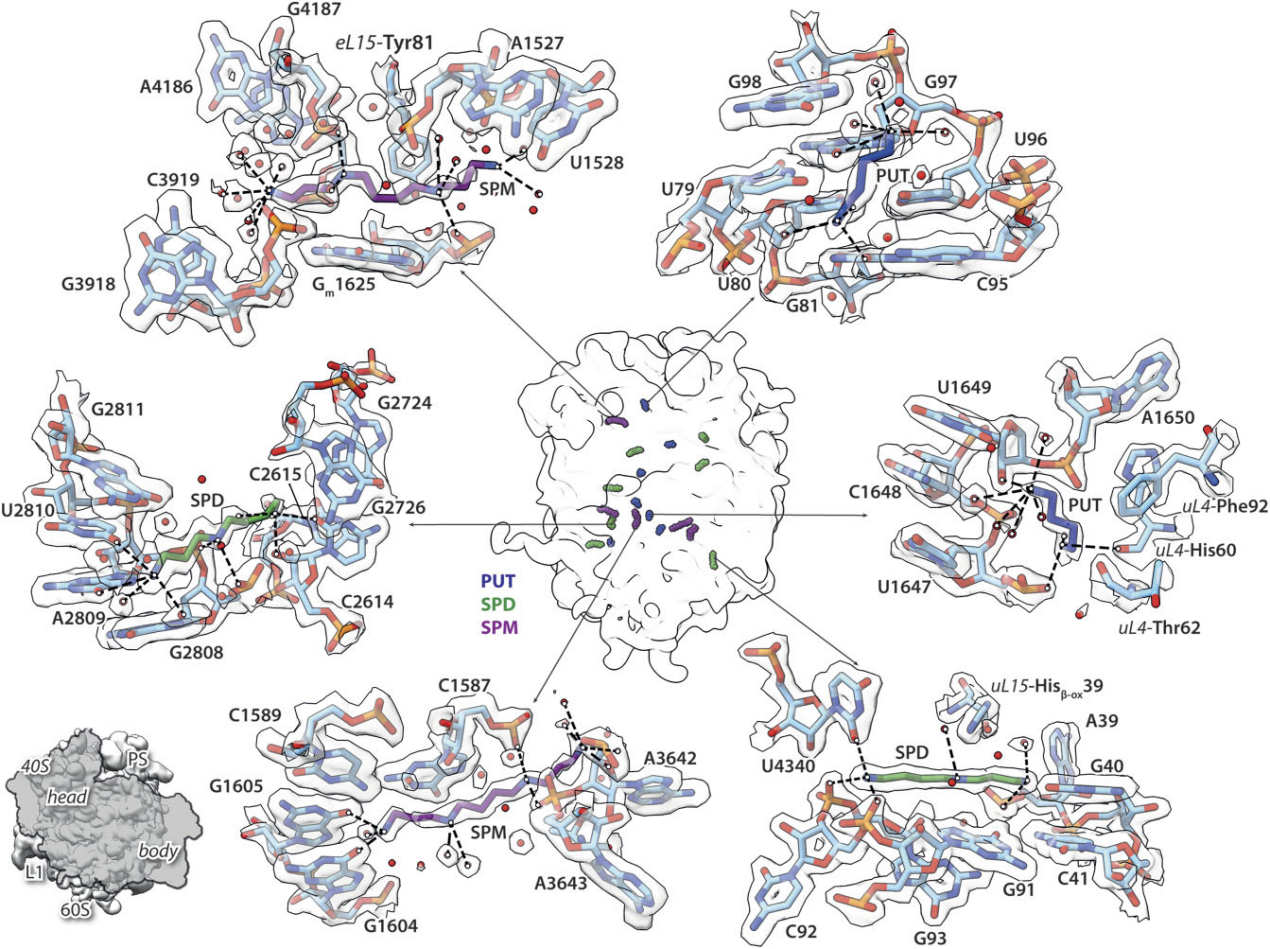
Polyamine-binding sites in the HeLa 60S ribosomal subunit. All identified binding sites of polyamines were mapped on the map of HeLa 60S ribosomal subunit shown in the centre as white density. Positions of seven molecules of PUT (blue), seven SPD (green) and five SPM (purple) are indicated. Close-up views, radially placed around the map, show two examples of each polyamine class as sticks surrounded by transparent density contoured at 8σ level. Bound water molecules are shown as red spheres and interactions are indicated by dashed lines.

The employed sample preparation method including sucrose in combination with high-quality cryo-EM data acquisition allowed us to achieve a global map resolution of 1.78 Å (FSC_0.143_) and local resolution in the 60S core hitting the Nyquist limit of 1.232 Å allowing to resolve atomic distances (Supplementary Figure S3 and Supplementary Table S1; decimated pixel size of the map at 0.616 Å). Based on the map, we could build a confident molecular model (Figure 1), unambiguously assign modifications in ribonucleotides (Supplementary Figures 7 and 8) and ribosomal proteins (Figures 2–4) and visualize the coordination of ligands like Mg^2+^ and K^+^ ions (Figure 5), metals and polyamines (Figure 6), as well as water molecules (Figures 4–6 and Supplementary Figure S9). Due to their flexible nature, the expansion segments and L1-stalk are less well resolved but can be partially visualized in maps filtered to 6 Å. A further variability analysis revealed more information about their dynamics, but did not yield any further atomic details (Supplementary Table S4 and Supplementary Figure S10). The map-to-model FSC_0.5_ of 1.87Å (Supplementary Figure S3C) illustrates that map and built model are in excellent agreement and that our modelling has fully exploited the potential of the cryo-EM map. This is on par with recent structures of the prokaryotic 70S ribosome of *E. coli* (2.04 Å map-to-model FSC_0.5_) (74), the structures of the eukaryotic 40S ribosomal subunit of HEK293 cells (2.12 Å map-to-model FSC_0.5_) (72) and the 80S ribosome of HEK Expi293F cells (1.78 Å map-to-model FSC_0.5_) (75) and HeLa cells (1.90 Å map-to-model FSC_0.5_) (76).

### Ribosome modifications

An indicator of high map quality is the appearance of aromatic moieties as rings, and RNA and protein modifications as additional densities on nucleotides and amino acids that can be discerned visually when interpreted against an unmodified model (Figure 3A and B). We set out to identify and describe all modifications contained in the HeLa 60S ribosomal subunit. We noticed that modifications cluster in the centre of the 60S ribosomal subunit, close the peptidyl transferase centre, and near the interface to the 40S ribosomal subunit (Figure 2). We found indications for three hitherto unknown rRNA modifications (Figure 2 and Supplementary Table S5). We were able to confirm 113 known rRNA modifications and four ribosomal protein modifications (Supplementary Table S5). Ten previously described modified nucleotides appeared unmodified, while further fourteen known modifications (9) could not be verified as they are expected in regions that could not be resolved in atomic detail. Additional methylation of eL29 Lys5 and eL42 Lys53 as recently described for the 80S from HEK293 cells (75) is not present in the HeLa 60S ribosomal subunit.

### Protein modifications

The most abundant protein modification identified in mammalian 60S ribosomal subunits is the oxygenation of histidine in β-position (22). HeLa 60S ribosomal subunits show two of these oxygenations, namely in ribosomal proteins uL2 at position His216 and uL15 at His39 (Figure 3A and B). The two hydroxyl groups display *2S, 3S*-stereochemistry in accordance with previous amino acid analyses (22). Oxygenation of uL15-His39 is facilitated by the Myc-induced-nuclear antigen (MINA, RIOX2). The residue is located about 11 Å from the E-site of the ribosome (28S rRNA G4371) and is involved in maintaining the 3′-end structural environment of the E-site-bound tRNA (45). Correct structural organization of the region neighbouring uL15-His39 is important for efficient translation elongation, as for instance antibiotic cycloheximide, which blocks translation, binds in a nearby pocket formed by 28S rRNA (H13, H82 and H88) and ribosomal proteins uL15, eL18 and eL42 by obstructing the binding site of the very 3′-adenylate of E-tRNAs CCA-end located between 28S G4371 and G_m_4370 [PDB-ID: 5LKS(77)].

Interestingly, in the HeLa 60S ribosomal subunits we find a SPD molecule filling a cavity, only 4 Å away from uL15- His_β-ox_ 39 (Figure 3A), that is also observed in HEK293 80S ribosomes (75). This is involved in water-mediated hydrogen-bonding network with the β-hydroxyl group (Figure 3A) which may have another stabilizing effect to the region. Recent work proposed for uL15-His_β-ox_39 an important role in efficient translation, especially of long mRNAs, which are translated by many ribosomes simultaneously (78). They detected changes in the pool of translated mRNAs in cells with ribosomes that contained only the non-hydroxylated mutant uL15-His39Ala, towards an increase in mRNAs with shorter coding sequences. This points to lower translation efficiency of these ribosomes that gives shorter and more abundant mRNAs an advantage in translation.

uL2-His_β-ox_216 is located close to the PTC and is facilitated by nucleolar protein 66 (NO66, RIOX1) (22). Previous work suggested that oxygenation of uL2-His216 is important for keeping H93 of the 28S rRNA in proper orientation to the peptidyl transferase centre, partially also due to a proposed additional hydrogen bond with *uL2*-His218 (79). Our structure shows that uL2-His_β-ox_216 is indeed nestled within the loop of H93. The oxygenation itself though does not seem to be involved in a direct contact with uL2-His218, which is >4 Å away, but stabilization could be mediated by participation of the β-hydroxyl group in an extended water-mediated hydrogen bonding network (Figure 3B). This network also involves 28S ribonucleotides G4541(O6) and G4545(O6) and spans towards Ψ_m_3818(O4) located closer to the PTC. This finding is supported by biochemical analyses in which region IV (28S ribonucleotides 4534–4548, H93), located close to the PTC, was protected against hydroxyl radical attack when bound to uL2-His_β-ox_216, but not to unmodified uL2-His216 (79). We conclude, that His_β-ox_216 positions the C-terminal loop of uL2 in a way that stabilizes H93, which then supports proper folding of the PTC.

Furthermore, we found His245 of uL3 to be methylated at position N_τ_ (Nε2) (Figure 3C). This modification was biochemically described for HEK293T cells (23,80) and HeLa cells transiently transfected with the methyl transferase METTL18 (23), whose activity was mapped to the nucleolus, indicating that this modification takes places during 60S ribosomal subunit assembly. The methylation was confirmed by cryo-EM for 80S ribosomes from naïve HEK293T cells [PDB-ID 6Y6X, (81)]. Conversely, 60S ribosomal subunits from METTL18KO HEK293T cells lacked the methylation [PDB-ID 7F5S, (80)]. τ-N-methylation disrupts the hydrogen bond of His245 (Nε2) with G1595 (O6) of 28S rRNA, which is in the loop of helix 35 in about 20 Å distance from the PTC, lining the nascent peptide tunnel. Genome-wide ribosome profiling and *in vitro* translation assays revealed that τ-N-methylhistidine on uL3 slows down translation elongation at Tyrosine codons (Tyr in the A-site), which in turn increases protein quality of Tyrosine-rich proteins in HEK293T cells (80). In other words, missing uL3-methylation induces the accumulation of unfolded and ultimately aggregated proteins in cells. His245 lies in the ‘basic thumb’ region (Arg234-Arg246) of uL3, which bridges the interaction of 28S rRNA helices H61, H73, and H90 and enables the formation of a dynamic ‘aminoacyl–tRNA accommodation corridor’ (82). The latter may be allosterically influenced by τ-N-methyl-His245 of uL3, leading to restricted degrees of freedom for tyrosinyl–tRNA in the A-site.

Lastly, we identified another protein methylation site in the HeLa 60S ribosomal subunit, namely at the ε-amine of Lys98 in eL40 (Figure 3D). eL40 was shown to be ubiquitinylated in yeast (83,84). Located at the surface of eL40 and the 60S ribosomal subunit and thus being solvent exposed, Lys98 represents a possible ubiquitination site, making its masking by methylation a possible regulatory mechanism to prevent mono- or polyubiquitination, especially in mature ribosomes. Furthermore, Lys98 is in close vicinity to the Cys_4_-zinc finger of eL40. Cys_4_-zinc fingers bind to nucleic acids, pointing to a possible role of methylation in the regulation of eL40-rRNA interactions during mammalian ribosome assembly. eL40 enters the maturing pre-60S very late in assembly, namely at stage III in the cytoplasm and is a prerequisite for uL16 binding (85). First shown for yeast (83), eL40 is synthesized as a genetic fusion with an N-terminal ubiquitin (encoded by human gene *uba52*), like 40S ribosomal protein eS31, which facilitates their folding and enhances solubility (86). Furthermore, this genetic fusion strongly protects Ub-eL40 from ubiquitin-independent high-level cotranslational protein degradation (87). As in previous cryo-EM studies on the HeLa ribosome (12), and in contrast to the proposed trimethylation for rat liver ribosomes (88) and the recently determined 80S ribosome structures from HEK293 cells (75), we identified Lys98 to be monomethylated in HeLa 60S.

### RNA modifications

We were able to build and confirm 113 rRNA modifications, which correspond to ∼2% of all encompassed ribonucleotides in this structure (Supplementary Table S5). Most of them are in the 28S rRNA, while three are found in the in the 5.8S rRNA, and none in the 5S rRNA. The most abundant modifications are pseudouridinylations (accounting for 48) and 2′-methylations of the ribose (accounting for 59). They are scattered throughout the whole 60S ribosomal subunit (Figure 2) and are thought to facilitate its structural integrity as they allow for enhanced interactions patterns stabilize the rRNA (89). Modifications of nucleobases (Figure 3E–J) cluster around the peptidyl transferase centre and the interface to the 40S ribosomal subunit (Figure 3).

#### Nucleobase modifications

We confirmed five known nucleobase modifications, which are all methylations facilitated by enzymes playing a role during 60S ribosomal subunit assembly (90,91), and could visualize their interaction networks in the three-dimensional context of the native 60S subunit.

Ribonucleotide A1322 (*m^1^*A1322) is methylated by the nucleolar factor NML (nucleomethilin), which plays a decisive role during subunit assembly and was shown to depend on glucose levels in the cell (92). The N1-methylation of A1322 (located in helix 25a) enhances interactions with the backbone phosphate between ribonucleotides U4445 and U4446 (located between helices 89 and 90) and increases the stacking ability to A3876 (located in helix 72) (Figure 3H), thereby stabilizing the interaction between distant rRNA sequences that come close together during rRNA folding in 60S ribosomal subunit assembly. The four mentioned ribonucleotides form the contact between domain 0 (A1322 and A3876) and domain V (U4445-U4446) of the mature 28S rRNA in the 60S ribosomal subunit, a known ‘modification hotspot’ at the interface of domain 0, II and V. In similar fashion *m^6^*A4220 exhibits enhanced stacking to ribonucleotides A4219 and G4222 (helix 81, domain V), as well as an interaction with the ribose of U1863 (helix 39, domain II) (Figure 3I). A4220 is modified by the methyltransferase ZCCHC4 in the nucleolus, which was shown to positively affect 60S ribosomal subunit biogenesis and to increase 60S cellular levels (93,94). Together with METTL18, the methyltransferase for *uL3*-His245, ZCCHC4 has also been shown to ensure proper translation of the codons AAA, CAA, GAA which are especially dependent on modifications of wobble uridine modifications in their decoding tRNAs (23,94).

The 5-methylation of cytidines is quite common in RNAs (95). In the 28S rRNA it is present in two positions, namely *m^5^*C3782 and *m^5^*C4447 (Figure 3F and G). C3782 is methylated by methyltransferase NSUN5, which is required for maintaining productive global protein synthesis (96). The 5-methylation of C3782 provides additional stability by enhancing base-stacking to ribonucleotides C3781 and G3811. *M^5^*C4447 is methylated by the related transferase NSUN1 (97,98). The methylation of universally conserved C4447 allows base pairing with A4396 – the PTC residue just next to the catalytic base A4397 – along the Hoogsteen edge, bridging the two RNA-strands (79). Further increase in local stability comes from enhanced stacking to G_m_4392, which itself is modified via ribose-O2’-methylation (Figure 3F).

While there are corroborations that the conserved modifications are essentially a quality control of the ribosomal assembly facilitated by the enzymes (90) our results suggest that some of the modifications themselves play a role in stabilizing parts of the PTC via enhanced interactions.

In addition, inspection of our map suggests a ‘new’ modification of 28S nucleotide C1859. It was modelled as N4-methylcytidine (*m^4^*C1859) as methylation is most likely, but from the density it cannot be discerned whether it might be alternatively an amination or different kind of modification. This modification was not found in biochemical studies and its function is unclear. Our map indicates that it could provide additional stability by enhancing base-stacking to ribonucleotides A1858 and Ψ1860. Also, it locates to the interface of 28S rRNA domains 0, II and V.

#### 2′-O-methylation of the ribose

The most common modification in the HeLa 60S ribosomal subunit is methylation of ribose at atom O2’. In human rRNA this type of methylation is facilitated by small nucleolar RNAs (snoRNAs) (99), that bind to specific complementary sequences on the substrate RNAs to form a guide-target duplex, where a target nucleoside is modified by RNA-binding proteins, serving as methyltransferases. In our structure, we were able to identify 59 of these modifications (Supplementary Figure S7). While most of them have been described in previous structural studies (9,12), we found density that could be interpreted as an additional ribose-methylation at G_m_3669 here for the first time (Figure 2). Therefore, it is unknown which snoRNA (SNORD) is mediating the methylation. We searched the snoDB via BLAST [RNAcentral (66)] and found a known complementary snoRNA in *Trypanosoma cruzei* (TB9C2C1), with high similarity to a recently found human IncRNA (novel transcript: ENSG00000256116). Still, this putative modification needs independent confirmation by biochemical methods.

2′-O-ribose methylation affects not only ribosome biogenesis but also ribosomal functions, including translational efficiency and fidelity (89). One of the identified 2′-O-ribose methyltransferases is fibrillarin [FBL; (89,100)]. Its transcription is repressed by p53, which in turn leads to a decrease of 2′-O-ribose methylation in rRNAs, impairing the translational function of ribosomes (101). Furthermore, FBL overexpression facilitates tumorigenesis and is associated with poor survival of cancer-affected individuals, implying that rRNA modification plays a crucial role in cancer development. Indeed, levels of m^1^A-modified nucleosides are elevated in the urine of individuals with cancer (102).

#### Pseudouridinylations

Pseudouridines are isomers of uridine. As such, they are difficult to identify in cryo-EM maps as the electron densities of both isomers are very similar and undistinguishable at common map resolutions. A crucial difference between both is the introduction of an additional imine in pseudouridine at position C5 (Supplementary Figure S8). Imine, being an electron acceptor, offers the possibility to form additional hydrogen bonds that may lead to a further stabilization of the rRNA (103). Pseudouridinylations are carried out in a site-specific fashion by H/ACAbox RNP complexes, consisting of one H/ACA snoRNA and the four core proteins, namely NOP10, NHP2, GAR1 and dyskerin (15). Pseudouridines have different interaction modes (Figure 4), with a common feature of a distance to the electron donor of <3.2 Å (Supplementary Table S6). Also, the electron donor lies in the same plane as the aromatic ring of pseudouridine. The additional hydrogen bond is formed either with a water molecule like for Ψ224 and Ψ1862 (Figure 4A and B), with the phosphate of a nearby nucleotide, as for Ψ2508, Ψ_m_3818 and Ψ1677 (Figure 4C–E) or with the 2′-hydroxide of the ribose, as for Ψ4293 and Ψ4493 (Figure 4F and G).

Most of the pseudouridines identified in this study agree with previous cryo-EM and mass spectrometry studies (9,12). In cases in which the RNA-chain is too flexible to allow for the identification of nearby water molecules, pseudouridines could not be directly identified from the cryo-EM map. This is the case for, e.g. the predicted pseudouridines in regions H69 and H71. Additionally, U1582 and U4636 of the 28S rRNA have been previously identified as pseudouridines (9,12). Their coordination pattern in our structure though makes a modification unlikely (Figure 4H–J), as the distances to possible electron donors are larger than 3.2 Å. Therefore, we propose that in HeLa 60S ribosomal subunits these nucleotides are unmodified uridines.

In contrast, we found a water molecule in hydrogen-bonding distance (3.1 Å) to what would be the C5 atom of uridine at position 224 of the 28S rRNA which might suggest that this could be pseudouridine (Ψ224), as well (Figure 4A).

### Ion coordination

Metal ions like Mg^2+^ and K^+^ were found bound throughout the HeLa 60S ribosomal subunit. As they are closely coordinated to ribonucleotides, partially with bond lengths similar to covalent bonds, high resolution is necessary to unambiguously identify them. Earlier cryo-EM work with global resolution of 2.9 Å erroneously interpreted continuous density, seemingly binding to nucleotides, as covalently bound acetyl- and propyl-modifications (12). Re-evaluation using electrostatic potential maps clarified this misinterpretation (104). In our map metal ions and water molecules appear as distinct globular features (Figure 5).

Like pseudouridines, a differentiation of metal ions in cryo-EM maps based on their electron density is nearly impossible. However, their chemical properties make them distinguishable. Mg^2+^ is the most abundant metal bound to the ribosome, where it coordinates to negatively charged phosphate groups of the ribose-phosphate-backbone. It also preferably forms octahedral complexes of the structure [Mg(II)(H_2_O)_n_X_6-n_]^Y^ (34), where X are other electron donors (Figure 5A–E). These can be either further water molecules, phosphates or in rare cases also the imines of nucleobases (Figure 5B). The distances between the central atom Mg^2+^ and its ligands are shorter than hydrogen bond distances and range from 1.8–2.4 Å. Mg^2+^-complexes are predominately found in proximity to the phosphate-backbone and stabilize RNA–RNA and RNA–protein interactions.

The ionic radius of K^+^ is slightly larger than of Mg^2+^, and K^+^ prefers to involve in pentagonal complexes of the form [K(H_2_O)_n_X_5-n_]^Y^ (34). The increased ionic radius goes along with longer coordination-bond lengths of 2.8–3.2 Å. K^+^ predominantly coordinates to water molecules and nucleobases (Figure 5F and G). The identified ion positions agree with recent high-resolution studies on the human 80S ribosome (72,75).

### Polyamines

Most polyamines in the cell are bound to RNA, and particularly their binding to ribosomes, mRNAs and tRNAs influences protein translation (105). In our structure, we identified 19 polyamine molecules. We assigned PUT, SPM and SPD according to the density. SPM and SPD were included in the sample preparation buffer at near-physiological concentrations (45). Five SPM and seven SPD molecules, respectively, were placed in the electron density. Due to the flexibility of polyamines, the molecules might be resolved only partially in the density map. Therefore, SPM and SPD might be misidentified as the shorter PUT. We identified and placed seven PUT in the structure, one of them being unambiguous. PUT was not added during preparation and therefore may originate from the HeLa cells or traces of PUT present in the used commercial SPM/SPD chemicals (Figure 6 and Supplementary Table S7). The polyamine ligands appear mainly anchored to the ribonucleotide phosphates but can also interact with many types of electron donors like waters or heteroarenes via the electron acceptor properties of the amines. In that way, they reside in water-filled cavities and are involved in large water-mediated interaction networks. Comparison with other recent cryo-EM ribosome structures (10,71,75) suggests, that water molecules and polyamines are somewhat interchangeable (Supplementary Figure S9 and Supplementary Table S7). While we included ions and polyamines at near-physiological concentrations (2 mM MgCl_2_, 90 mM KCl, 0.5 mM SPD, 0.04 mM SPM), others used buffers with higher Mg^2+^ content without polyamines [5 mM MgCl_2_, 100 mM KCl; PDB-ID: 8A3D (71)] or higher salt and polyamine concentrations [5 mM MgCl_2_, 10 mM NH_4_Cl, 140 mM KCl, 2 mM SPD and 5 mM PUT; PDB-ID: 8GLP (75)]. This results in differences in metal ion and polyamine distribution. However, a few positions, e.g. one about 30 Å away from the PTC (Figure 6, SPM bound to 28S rRNA nucleotide G4187, Supplementary Figure S9), show conserved binding of polyamines which can either be SPMs or SPDs. This binding site seems to be also found in other eukaryotic ribosomes like the one from tobacco plant, suggesting preservation of it among species. Interestingly, our structure and the one from tobacco plant [PDB-ID: 8AZW (10)] using the same polyamine buffer, show the highest similarity in polyamine pattern further emphasizing buffer dependency (Supplementary Table S7). In some cases, differences between the polyamine binding in 60S from HeLa cells and from plant ribosome can be derived from the structure as e.g. the SPD close to 28S-U291 is absent in plants as there 25S-A47 exhibits a different conformation and occupies this site.

### Tentative evaluation of occupancy possible

Our cryo-EM map is the consensus of >800 000 LSUs, which are not necessarily identical. Therefore, it describes the congruent state of these single events. In case that a modification appears only at lower contour level of the EM map compared to neighbouring atoms, this might be a result of the modification being present in a significant fraction of the sample, but not ubiquitous. This might be the case for *m^4^*C1859 (Figure 3E), where at 5.5σ-level there is a clear sign of a modification, possibly a methylation, at N4, while at higher contouring level of the EM map this additional density seems to disappear. This happens even though the neighbouring atoms are well resolved and there is no indication of a flexibility of this modification. While we are not able to assign percentages as possible in mass spectrometry studies (9), we can suggest simultaneous occurrence of modified and unmodified C1859 in the sample.

In summary, in this study we present the high-resolution structure of the HeLa 60S ribosomal subunit. The excellent global resolution of 1.78 Å allowed us to independently identify and specify RNA and protein modifications and to build an atomic model including extensive polyamines, water molecules and ions. Independent of biochemical data, we discovered three putative previously unknown modifications, and were able to explain stabilizing effects of water-mediated hydrogen-bonding networks which could not be revealed by other experimental methods.

This work shows the capacity of cryo-EM for *de novo* identification of diverse features of macromolecular structures, given a high-enough map resolution, simultaneously integrating previous biochemical work and going hand-in-hand with an integrative approach, including biochemical methods to substantiate map-based findings.

## Supplementary Material

gkae1191_Supplemental_File

## Data Availability

Detailed information for all maps and models generated in this work is provided in Supplementary Table S1 and Supplementary Table S2. The map from single-particle reconstruction has been deposited in the Electron Microscopy Data Bank (EMDB) under accession code: EMD-18765 and the atomic model in the Protein Data Bank (PDB) under accession code: 8QYX. Tilt series are available from the authors upon request.

## Notes

Present address: Jörg Bürger, Institute of Chemistry and Biochemistry, Core Facility BioSupraMol, Freie Universität Berlin, Fabeckstr. 36a, 14195 Berlin, Germany.
