# Supplementary material for: Visualizing the modification landscape of the human 60S ribosomal subunit at close to atomic resolution
Source: Nucleic Acids Res. 2024 Dec 10;53(1):gkae1191. doi: 10.1093/nar/gkae1191 (PMC11724314; doi:10.1093/nar/gkae1191)
Supplement: gkae1191_Supplemental_File [file gkae1191_supplemental_file.pdf]

SUPPLEMENTARY FIGURES AND TABLES

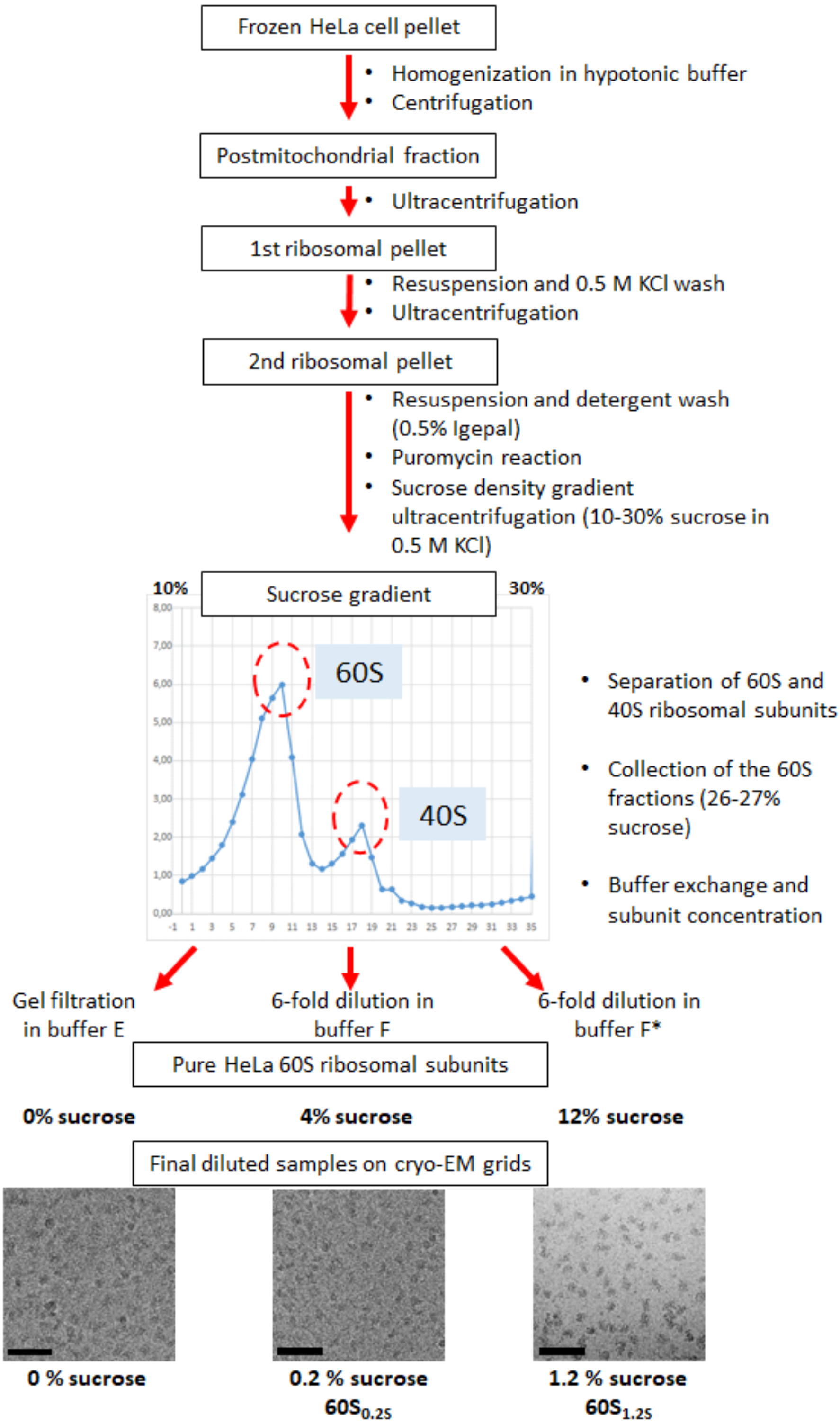

Supplementary Figure 1: Workflow of the 60S ribosomal subunit purification and grid preparation. 60S ribosomal subunits were prepared from frozen HeLa cell pellets using centrifugation and ultracentrifugation steps followed by separation via a sucrose density gradient. The purified subunits were stored in three different buffer conditions containing sucrose in concentrations ranging from 0% to 12%. Prior to grid preparation, the samples were diluted in sucrose-free buffers. Resulting electron micrographs show various degrees of aggregation depending on sucrose concentration in the samples. The defocus values for the EM micrographs are in the range of -2 µm. Scale bars represent 100 nm.

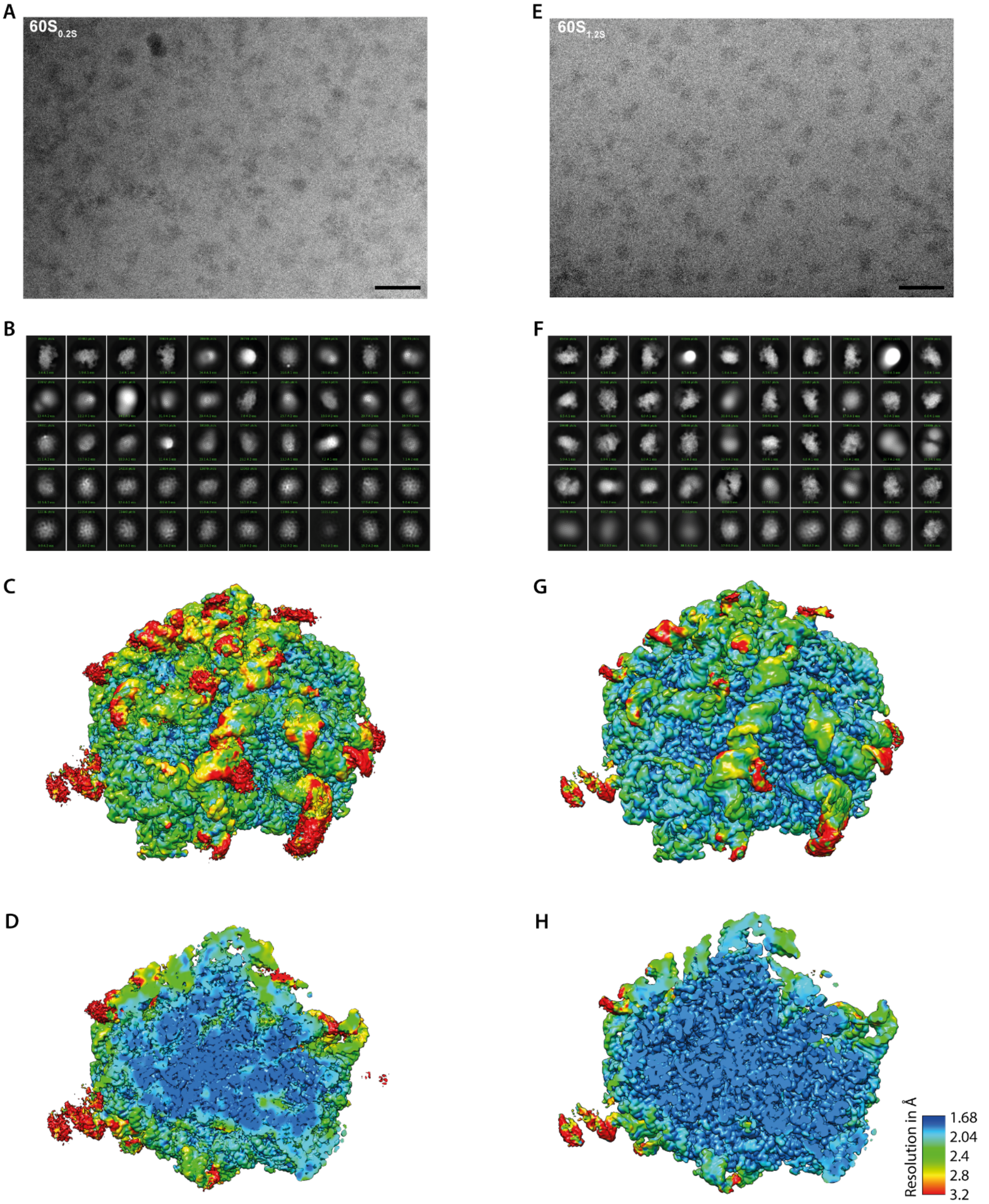

Supplementary Figure 2: Sucrose concentration in the sample influences 60S ribosomal subunit integrity. Comparison of cryo-EM datasets and single-particle 3D reconstructions of HeLa 60S ribosomal subunits vitrified in the presence of 0.2% sucrose **(A-D)** and 1.2% sucrose **(E-H)**. The data sets underwent identical processing and analysis, both resulting in maps with a global resolution of 2.04 Å ($FSC_{0.143}$). **(A,E)** Representative micrographs taken at -1.8 µm defocus showing 60S ribosomal subunit particles. Scale bars represent 50 nm. **(B,F)** 2D class averages from all picked particles classified in 50 classes. $60S_{0.2S}$ particles classified into five "shiny" classes with 22.5% of the particles accumulating to these. In comparison, $60S_{1.2S}$

particles yielded 39 "shiny" classes encompassing 70.2% of the picked particles. **(C,G)** Local resolution maps in crown view with focus on expansion segments. **(D,H)** Central slice through maps in **(C)** and **(G)**. The threshold of the maps was adjusted to show a comparable level of volume in both datasets. The local resolution is color-coded from high resolution in blue (Nyquist-limit 1.648 Å) to lower resolution red (3.2 Å).

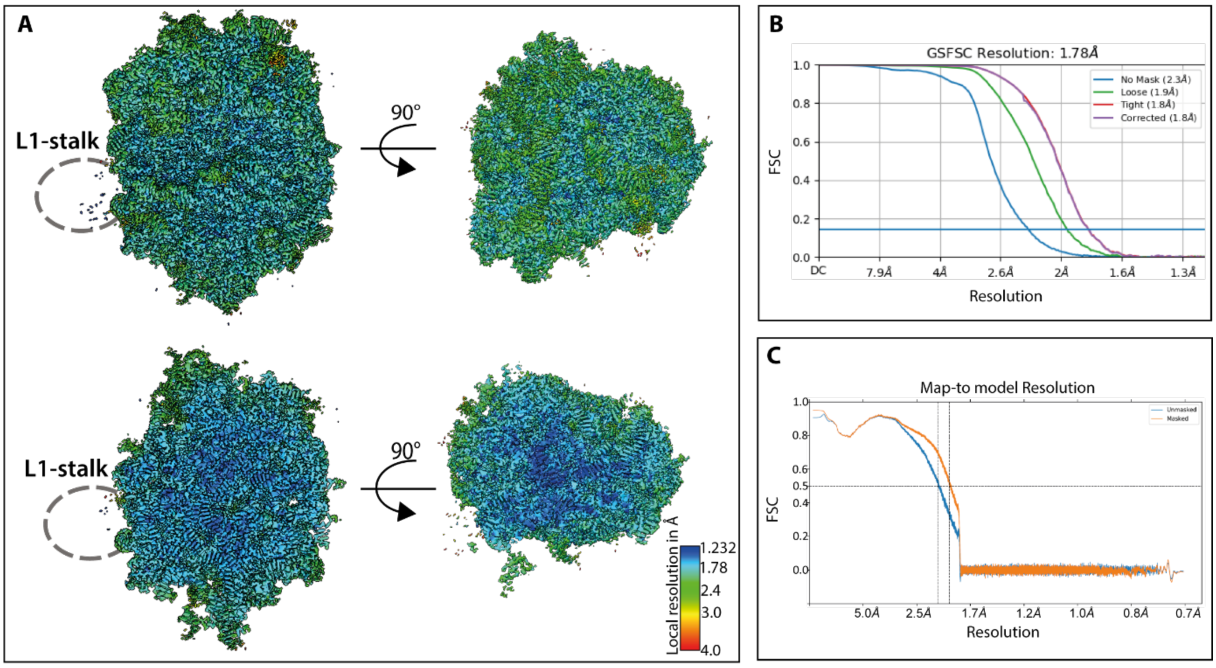

Supplementary Figure 3: Resolution estimation of the 60S ribosomal subunit single-particle reconstruction. **(A)** Local resolution maps of the $60S_{1.2S}$ 3D reconstruction. Top panel: View on the 40S interface of the 60S subunit (left) and the crown-view (right). Bottom panel: Central slice through the map with the same views as above. The resolution limits are color-coded from high resolution in blue (Nyquist-limit 1.236 Å) to lower resolution in red (4.0 Å). **(B)** Fourier shell correlation (FSC) curves from the final refinement in CryoSPARC (1) indicating a global resolution of 1.78 Å ($FSC_{0.143}$) for the $60S_{1.2S}$ 3D reconstruction. **(C)** Map vs. model cross-correlation FSC curves from validation in phenix.validation_cryoem (2) indicating a resolution of 1.87 Å ($FSC_{0.5}$).

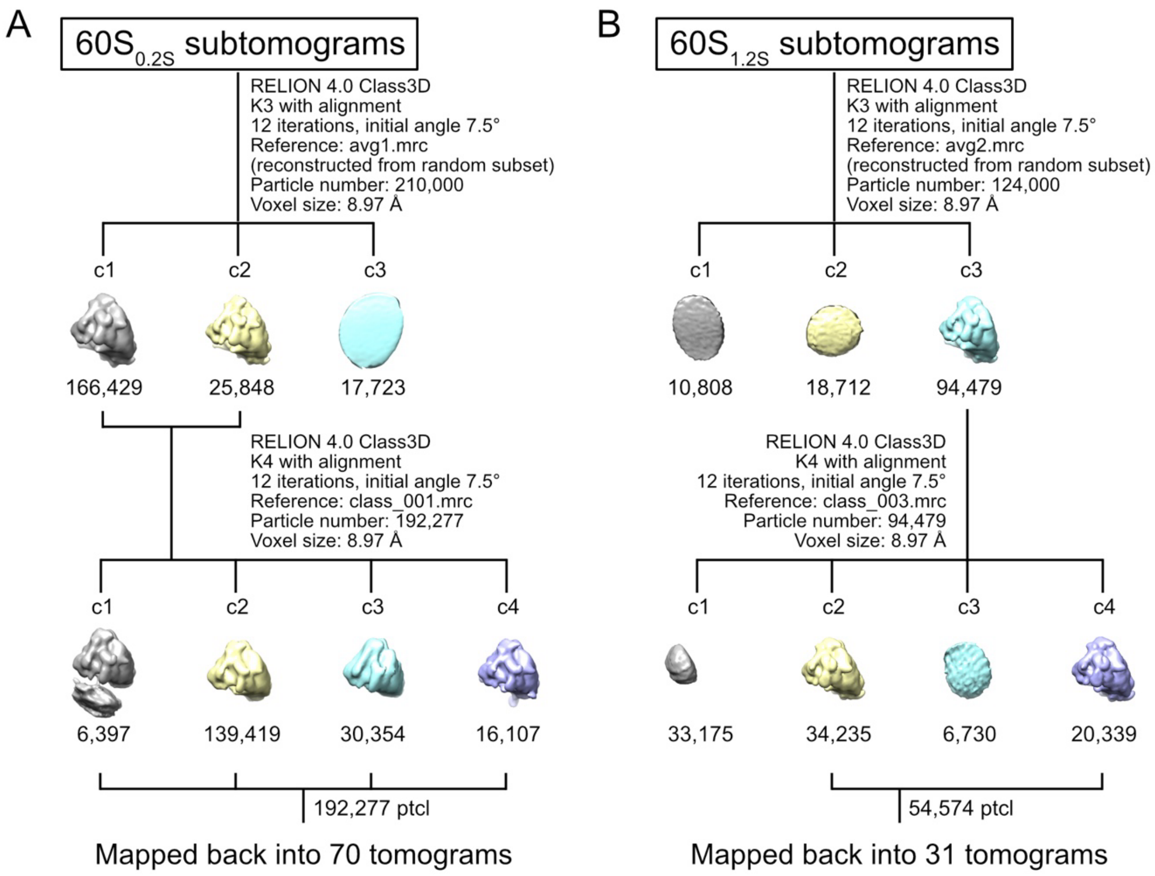

Supplementary Figure 4: Sorting scheme of 60S ribosomal subunit subtomograms. After template matching in Warp (3), subtomograms of $60S_{0.2S}$ **(A)** and $60S_{1.2S}$ **(B)** were extracted at 8.97 Å voxel size (bin 4) and classified in 3D using RELION 4.0 (4), to remove false positive picks. The final particle coordinates were mapped back into the tomograms (Supplementary Figure 5).

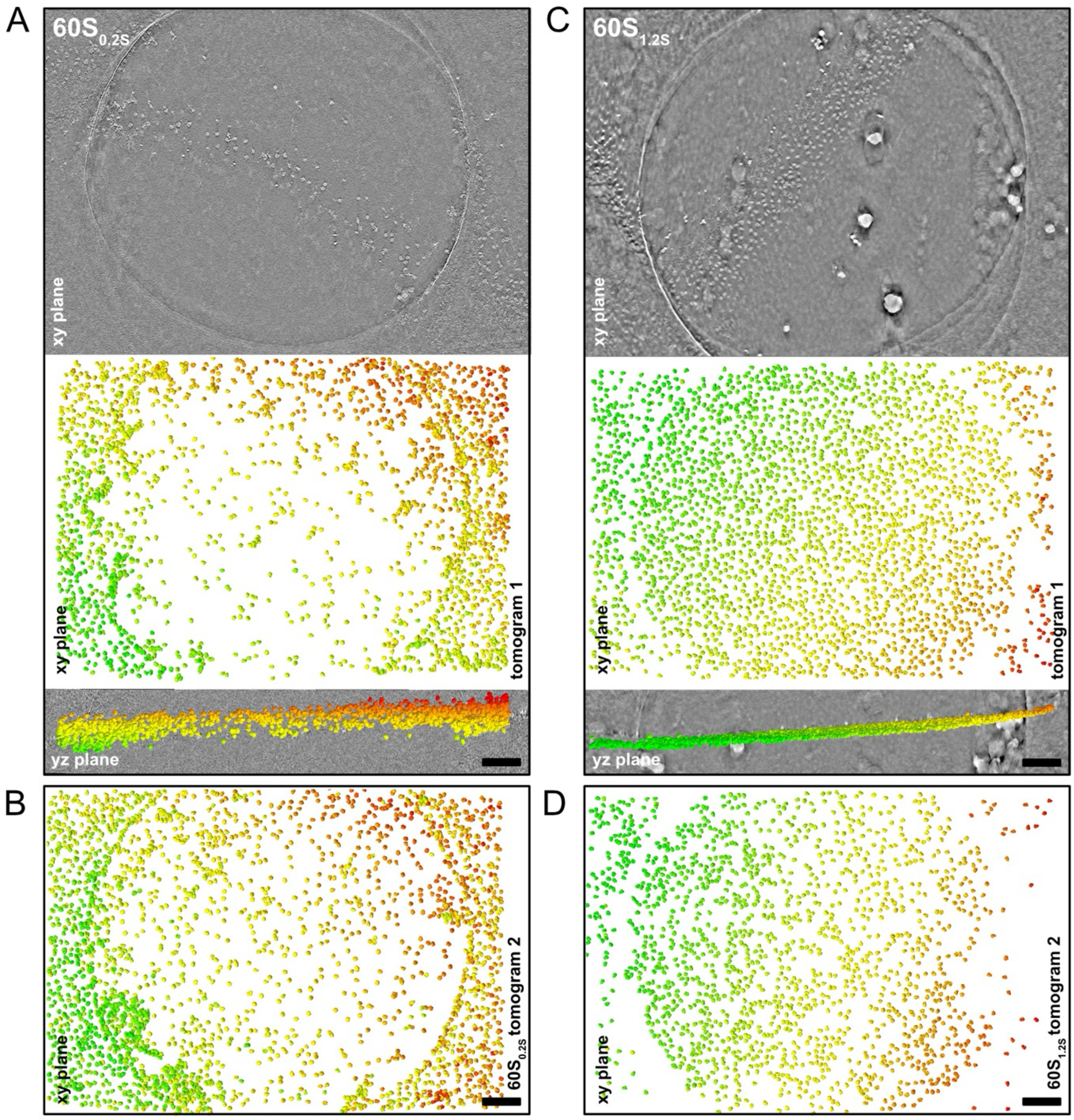

Supplementary Figure 5: 60S ribosomal subunit distribution in vitreous ice. Tomographic reconstruction of 60S ribosomal particles vitrified in the presence of 0.2% sucrose **(A,B)** and 1.2% sucrose **(C,D)**. The particle distribution above two representative carbon holes per condition is shown in xy plane **(A-D)**. Particle coordinates of cleaned subtomograms were mapped back into the tomograms using the ArtiaX plugin (5) in ChimeraX v1.7 (6) and coloured according to their position in the z-dimension (from green to red). In addition, for one tomogram per condition, the central slices through a deconvolved version of the tomogram in xy plane and yz plane are shown **(A,C)**. Scale bars represent 200 nm.

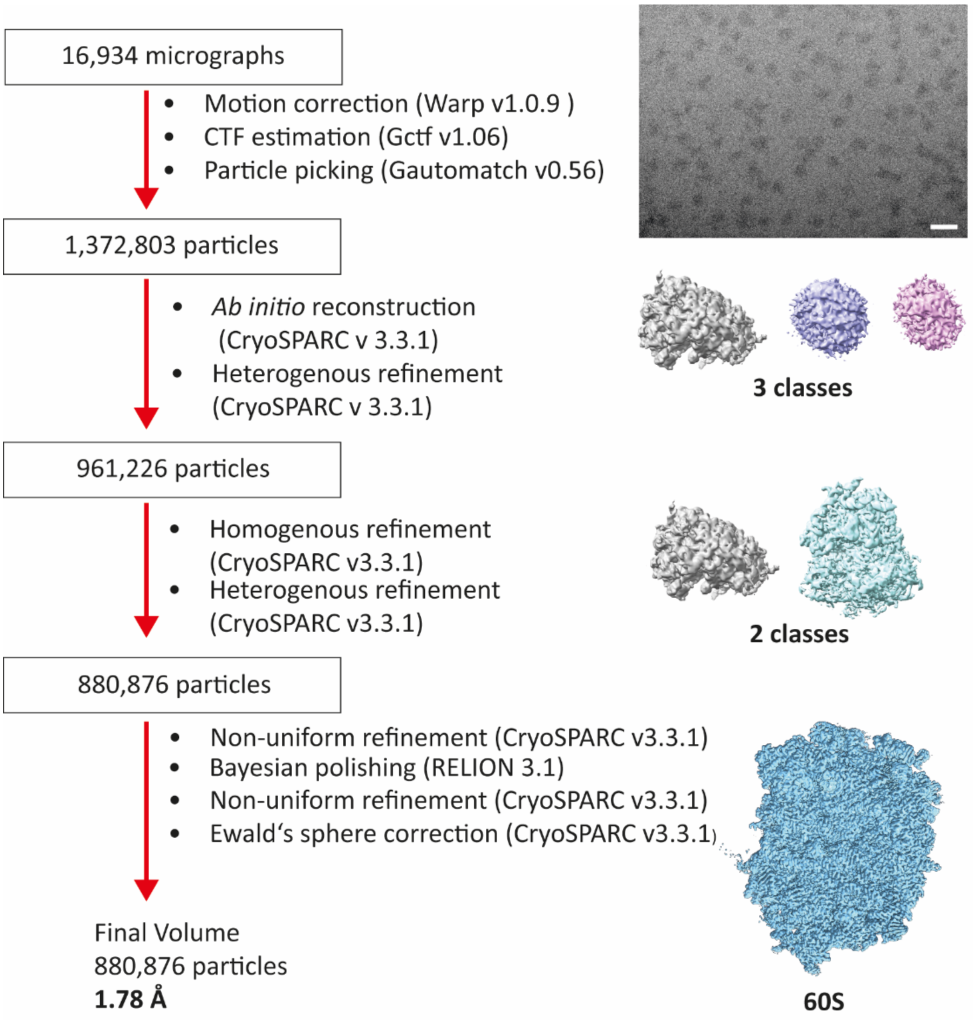

Supplementary Figure 6: Processing scheme for the single-particle reconstruction of the 60S ribosomal subunit. Initial motion correction was performed with Warp (3), followed by CTF estimation using Gctf (7) and particle picking in Gautomatch (8). A resulting representative micrograph is shown. Particle images were extracted in RELION 3.1 (9) and imported to CryoSPARC v3.3.1 (1). After *ab initio* reconstruction the particles were subjected to consecutive hetero- and homogenous refinement to remove non-ribosomal particles and 80S ribosomal particles (initial volumes used for hetero-refinement are shown). $60S_{1.2S}$ ribosomal subunit particles were further refined using non-uniform (NU) refinement (correcting for higher order aberrations) and CTF-per particle refinement. The refined particles were exported to RELION 3.1 for Bayesian polishing. Polished particles with a pixel size of 0.618 Å were re-imported to CryoSPARC for a final NU refinement and reconstruction with Ewald's sphere correction, which improved the global resolution of the density map to 1.78 Å ($FSC_{0.143}$). Scale bar in micrograph represents 50 nm.

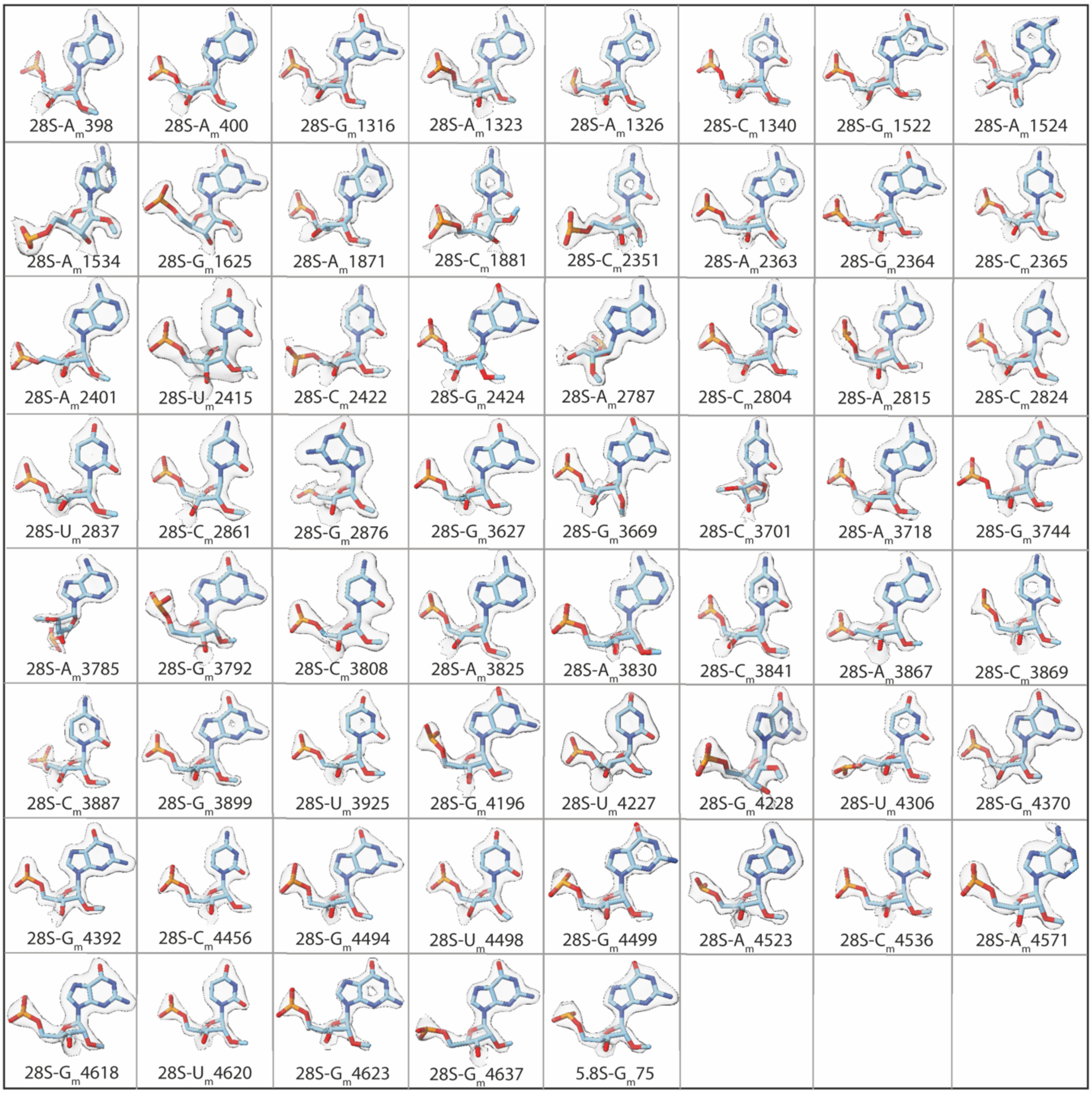

Supplementary Figure 7: Close-up views of O2′-methylated nucleotides in the 60S ribosomal subunit. Modified nucleotides are shown as sticks with transparent density adjusted to 5-14 σ-levels for similar visual impression.

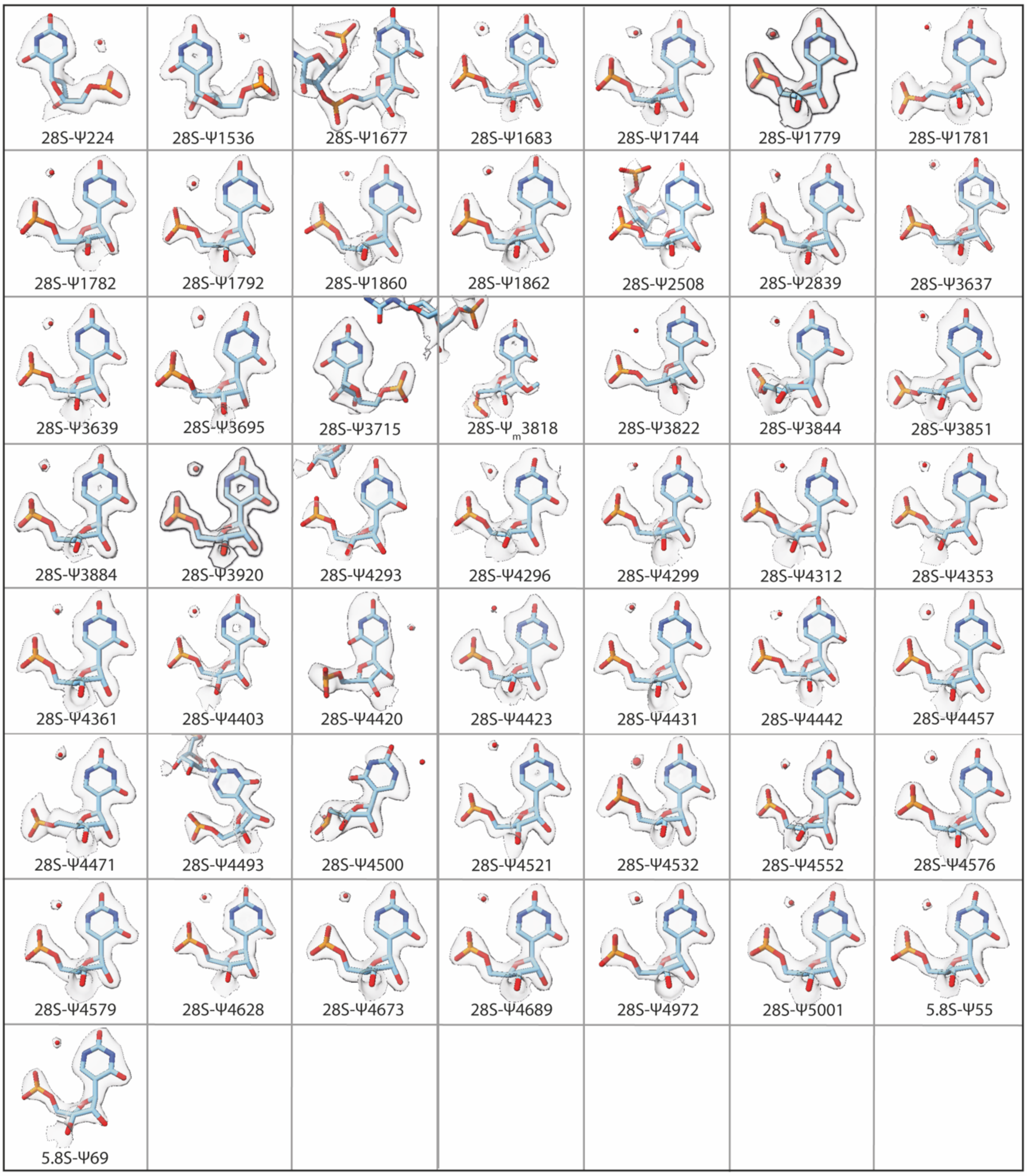

Supplementary Figure 8: Close-up views of identified pseudouridines and imide interaction partners in the 60S ribosomal subunit. Pseudouridines are shown as sticks, water molecules as red dots with surrounding transparent density adjusted to 5-14 σ-levels for similar visual impression.

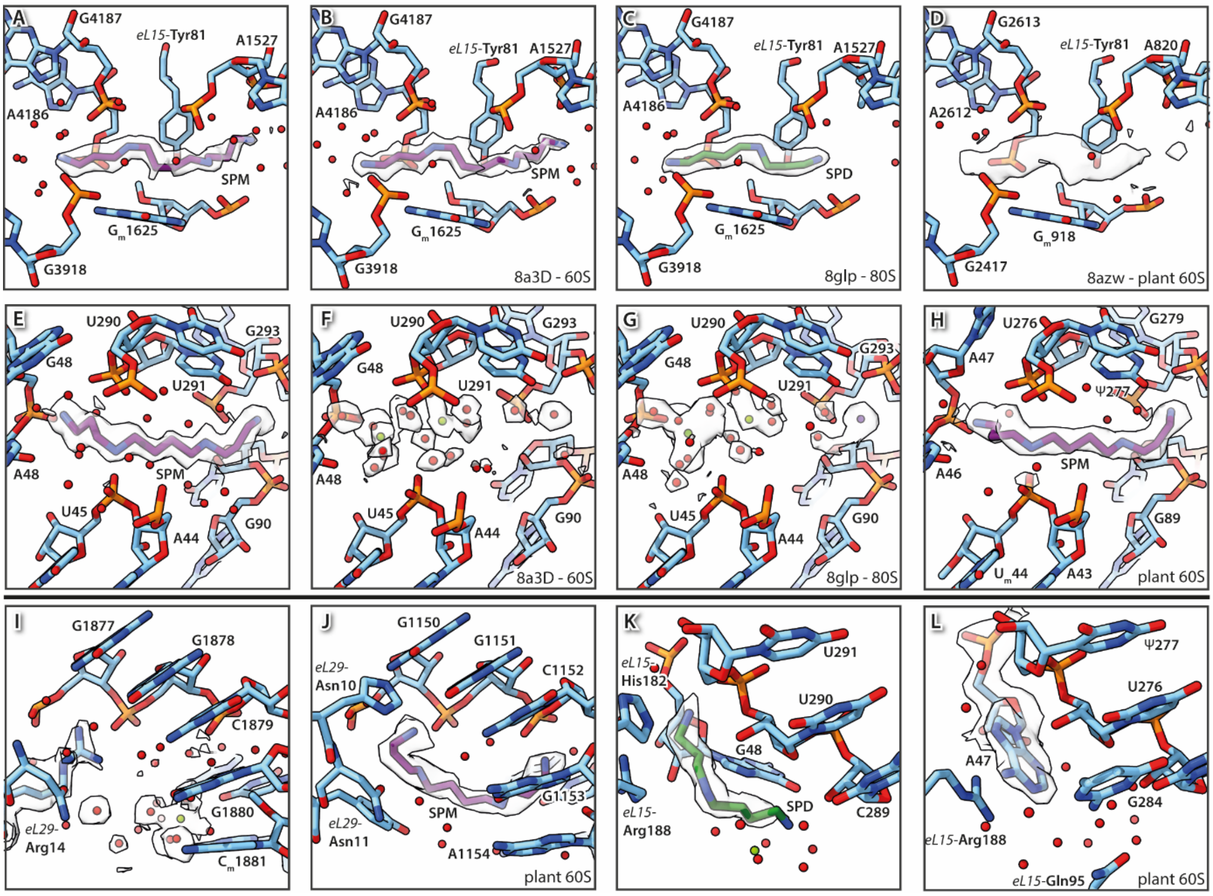

Supplementary Figure 9: Comparison of exemplary polyamines in high-resolution structures of 60S ribosomal subunits. **(A)** Spermine close to G_m1625 of 28S rRNA in 60S from HeLa cells (this work). **(B)** The same position in 60S from HEK cells with spermine bound [PDB-ID: 8A3D, (10)] and **(C)** 80S from HEK cells with spermidine bound [PDB-ID: 8QLP, (11)]. **(D)** No polyamine was built into the density found at the same position in 60S from tobacco plant [PDB-ID: 8AZW, (12)]. **(E)** Spermine was found close to nucleotide G46 of 28S rRNA in 60S from HeLa cells (this work). **(F, G)** The same position in 60S from HEK cells and 80S from HEK cells with water and Mg^{2+} binding there and **(H)** spermine binding instead in 60S from tobacco plant. **(I)** Mg^{2+} and water binding to 60S from HeLa cells (this work) at loop HS41 with el29-Arg14 closely associated. **(J)** In plant 60S spermine is binding there with el29-Asn10 close by. **(K)** Spermidine binding to H21 in the here presented structure and nucleotide G48 of 28S rRNA pointing away compared to **(L)** the plant 60S showing a different conformation of nucleotide A47 at this position and no polyamine binding. Nucleobases and peptides are shown as sticks, water molecules as red spheres and magnesium ions as green spheres. Transparent electron density at a contour level of 5.5 σ is shown.

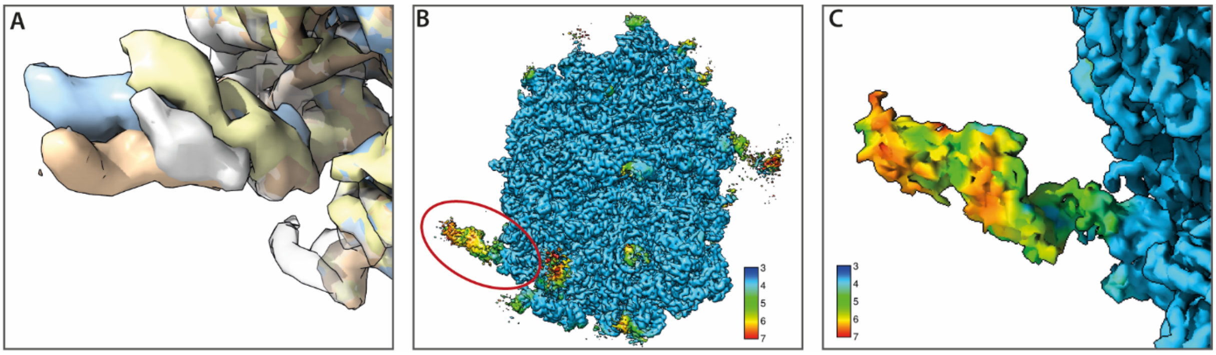

Supplementary Figure 10: Impressions from the 3D variability analysis for ES39. (A) Close-up views of four distinct states of expansion segment ES39, each comprising ~30k particles of the original 880k particle set are shown colored in light blue (state 1), peach (state 2), yellow (state 3) and white (state 4), respectively. State 4 also shows an enhanced density for parts of neighboring ES7 (B) Local resolution map for state 1 shows density for the previously "missing" structure at a magnitude below the rest of the map. (C) Close-up view of the state 1 in (B) shows clear helical conformation with the map being at 5-6 Å resolution.

Supplementary Table 1. Data collection and refinement statistics

Data Collection	8QYX<br>EMD-18765
Magnification	105,000x
Voltage (kV)	300
Electron dose (e^-/Å^2)	49
Nominal defocus range (µm)	-0.5 to -2
Pixel size (Å/px)	0.412
Map sharpening B factor (Å^2)	38.3
Resolution (Å, 0.143 FSC)	1.78
Symmetry imposed	C1
Initial particle images (no.)	1,370,000
Final particle images (no.)	880,818
Final map pixel size (decimated, Å/px)	0.616
**Model refinement**	
Initial model used (PDB ID)	6EKO
Model resolution (Å)<br>FSC threshold = 0.143	1.80
Model resolution (Å)<br>FSC threshold = 0.5	1.86
Model vs. map correlation coefficient (cc_mask)	0.83
Model composition	
Non-hydrogen atoms	150,022
Protein residues	6,429
RNA residues	3,986
Water	11,854
Ligands	235
B factors (Å^2) (mean)	
Protein	17.78
RNA	26.34
Water	14.32
Ligands	13.04
**Validation (proteins)**	
Poor rotamers (%)	0.13
Ramachandran outliers (%)	0.03
Ramachandran favored (%)	98.40
Bad backbone bonds (%)	0.0
Bad backbone angles	0.0
**Validation (nucleic acids)**	
Correct sugar puckers (%)	78
Good backbone conformations (%)	83
**Clash score, all atoms**	4.75
**Molprobity score**	1.24

Supplementary Table 2. Data collection and processing statistics tilt series

**Data Collection**	**$60S_{0.2S}$**	**$60S_{1.2S}$**
Magnification	19,500x	19,500x
Voltage (kV)	300	300
Camera	K3	K3
Energy filter slit width (eV)	20	20
Objective aperture (µm)	100	100
Movie frames per tilt	10	10
Target dose rate on camera (e^-/px/s)	8.4	7.8
Electron dose tilt series (e^-/Å^2)	109	102
Tilt range (°)	±48 (dose symmetric)	±48 (dose symmetric)
Tilt increment (°)	3	3
Defocus range (µm)	-3 to -7	-3 to -7
Pixel size (Å)	2.2425	2.2425
**Data processing**		
Symmetry imposed	C1	C1
Subtomograms extracted (no.)	210,000	124,000
Subtomograms final (no.)	192,277	54,574
Subtomogram voxel size (Å)	8.97	8.97

Supplementary Table 3. Relative ice thickness of representative tomograms

Tomograms 60S 0.2% sucrose	Thickness (nm)			Mean value (nm)	Deviation (nm)
MGS001_T2_ts_001.mrc	60	62	65	62.3	2.5
MGS001_T2_ts_002.mrc	58	57	60	58.3	1.5
MGS001_T2_ts_003.mrc	46	48	49	47.7	1.5
MGS001_T2_ts_004.mrc	47	48	47	47.3	0.6
MGS001_T2_ts_005.mrc	50	51	52	51.0	1.0
MGS001_T2_ts_006.mrc	50	51	52	51.0	1.0
MGS001_T2_ts_007.mrc	50	52	49	50.3	1.5
MGS001_T2_ts_008.mrc	44	45	52	47.0	4.4
MGS001_T2_ts_009.mrc	47	74	51	57.3	14.6
MGS001_T2_ts_010.mrc	45	50	45	46.7	2.9
MGS001_T3_ts_002.mrc	48	50	55	51.0	3.6
MGS001_T3_ts_003.mrc	48	43	122	71.0	44.2
				53.4	6.6
**Tomograms 60S 1.2% sucrose**	**Thickness (nm)**			**Mean value (nm)**	**Deviation (nm)**
MGS001_T1_ts_001.mrc	48	50	47	48.3	1.5
MGS001_T1_ts_002.mrc	48	37	45	43.3	5.7
MGS001_T1_ts_003.mrc	53	51	49	51.0	2.0
MGS001_T1_ts_004.mrc	51	51	56	52.7	2.9
MGS001_T1_ts_005.mrc	43	46	53	47.3	5.1
MGS001_T1_ts_006.mrc	45	56	49	50.0	5.6
MGS001_T1_ts_007.mrc	44	46	49	46.3	2.5
MGS001_T1_ts_008.mrc	53	52	50	51.7	1.5
MGS001_T1_ts_009.mrc	52	50	53	51.7	1.5
MGS001_T1_ts_010.mrc	50	53	50	51.0	1.7
MGS001_T1_ts_011.mrc	50	44	48	47.3	3.1
MGS001_T1_ts_012.mrc	48	52	52	50.7	2.3
				49.3	3.0

Supplementary Table 4: Overview of *in silico* 3D variability and sorting results of selected variable regions

Structural element	Description of computationally isolated states
**L-Stalk**	improved density in three different orientations with 140k particles, 231k particles and 235k particles, local resolution >6 Å
**ES7**	very heterogenous and seemingly only partially ordered at best, no distinctive states found
**ES27**	isolated three helical states with 55k, 29k and 60k particles
**P-Stalk**	seems disordered, sorting led to no improvement
**ES5**	two different helical orientations, not well defined with 250k particles each
**ES31**	seems disordered, sorting led to no improvement
**ES39**	helical, with different orientations, sorting in distinct states (~30k particles) improved to 5 Å local resolution for a subset

Supplementary Table 5. Comparison of rRNA modifications

Chain	Position	This study	Taoka *et al.* (14)	Holm *et al.* (11)	Faille *et al.* (10)	Smirnova *et al.* (12)	Natchiar *et al.* (13)
**Organism cell line**		Human HeLa	Human HeLa	Human HEK Expi293F	Human HEK Expi293F	Tobacco plant	Human HeLa
**28S**	224	Ψ224					
**28S**	237						xp^6G237
**28S**	373						Gm373
**28S**	398	Am398	Am398	Am398	Am398		Am398
**28S**	400	Am400	Am400	Am400	Am400		
**28S**	729						m^2G729
**28S**	978						m^2G978
**28S**	1316	Gm1316	Gm1316	Gm1316	Gm1316		Gm1316
**28S**	1322	m1A1322	m1A1322	m1A1322	m1A1322	1mA656	m^1A1322
**28S**	1323	Am1323	Am1323				
**28S**	1326	Am1326	Am1326	Am1326	Am1326	Am660	Am1326
**28S**	1340	Cm1340	Cm1340	Cm1340	Cm1340	Cm674	
**28S**	1348						xp^4U1348
**28S**	1456						m^3C1456
**28S**	1517						m^2G1517
**28S**	1522	Gm1522	Gm1522	Gm1522	Gm1522	Gm815	Gm1522
**28S**	1524	Am1524	Am1524	Am1524	Am1524	Am817	Am1524
**28S**	1534	Am1534	Am1534	Am1534	Am1534	Am827	Am1534
**28S**	1536	Ψ1536	Ψ1536	Ψ1536	Ψ1536	Ψ829	
**28S**	1574						xp^6G1574
**28S**	1582		Ψ1582	Ψ1582	Ψ1582		Ψ1582
**28S**	1605						m^7G1605
**28S**	1625	Gm1625	Gm1625	Gm1625	Gm1625	Gm918	Gm1625
**28S**	1659						xp^4U1659
**28S**	1677	Ψ1677	Ψ1677	Ψ1677	Ψ1677	Ψ970	Ψ1677
**28S**	1683	Ψ1683	Ψ1683	Ψ1683	Ψ1683	Ψ976	Ψ1683
**28S**	1744	Ψ1744	Ψ1744	Ψ1744	Ψ1744	Ψ1016	
**28S**	1760	ND	Gm1760		ND		
**28S**	1773	ND	Um1773		ND		
**28S**	1779	Ψ1779	Ψ1779	Ψ1779	ND		
**28S**	1781	Ψ1781	Ψ1781	Ψ1781	Ψ1781		
**28S**	1782	Ψ1782	Ψ1782	Ψ1782	Ψ1782		
**28S**	1792	Ψ1792	Ψ1792	Ψ1792	Ψ1792	Ψ1064	
**28S**	1797						xe^7G1797
**28S**	1859	m4C					
**28S**	1860	Ψ1860	Ψ1860	Ψ1860	Ψ1860	Ψ1133	$m^1Ψ1860$
**28S**	1862	Ψ1862	Ψ1862	Ψ1862	Ψ1862	Ψ1135	
**28S**	1866						m^3U1866
**28S**	1871	Am1871	Am1871	Am1871	Am1871	Am1144	Am1871

**28S**	1881	Cm1881	Cm1881	Cm1881			
**28S**	1883						Gm1883
**28S**	1909						xp^{7}G1909
**28S**	2050						Gm2050
**28S**	2297						xe^{7}G2297
**28S**	2351	Cm2351	Cm2351	Cm2351	Cm2351	Cm1448	
**28S**	2363	Am2363	Am2363	Am2363	Am2363	Am1460	Am2363
**28S**	2364	Gm2364	Gm2364	Gm2364	Gm2364	Gm1461	Gm2364
**28S**	2365	Cm2365	Cm2365	Cm2365	Cm2365		Cm2365
**28S**	2380						m^{6}G2380
**28S**	2401	Am2401	Am2401	Am2401	Am2401		Am2401
**28S**	2415	Um2415	Um2415	Um2415	Um2415		
**28S**	2422	Cm2422	Cm2422	Cm2422	Cm2422		Cm2422
**28S**	2424	Gm2424	Gm2424	Gm2424	Gm2424		Gm2424
**28S**	2508	Ψ2508	Ψ2508	Ψ2508	Ψ2508		Ψ2508
**28S**	2522						m^{7}G2522
**28S**	2632	Ψ2632	Ψ2632	Ψ2632	Ψ2632		
**28S**	2754						xp^{6}G2754
**28S**	2773						Gm2773
**28S**	2786						xp^{3}Cm2786
**28S**	2787	Am2787	Am2787	Am2787	Am2787		
**28S**	2804	Cm2804	Cm2804	Cm2804	Cm2804	Cm1862	Cm2804
**28S**	2815	Am2815	Am2815	Am2815	Am2815		
**28S**	2824	Cm2824	Cm2824	Cm2824	Cm2824		
**28S**	2837	Um2837	Um2837	Um2837	Um2837	Um1894	
**28S**	2835	A	Ψ2835		A		
**28S**	2839	Ψ2839	Ψ2839	Ψ2839	Ψ2839		
**28S**	2861	Cm2861	Cm2861	Cm2861	Cm2861		Cm2861
**28S**	2876	Gm2876	Gm2876	Gm2876	Gm2876		
**28S**	3627	Gm3627	Gm3627	Gm3627	Gm3627	Gm2127	
**28S**	3637	Ψ3637	Ψ3637	Ψ3637	Ψ3637	Ψ2137	
**28S**	3639	Ψ3639	Ψ3639	Ψ3639	Ψ3639	Ψ2139	
**28S**	3669	Gm3669					
**28S**	3695	Ψ3695	Ψ3695	Ψ3695	Ψ3695	Ψ2194	
**28S**	3701	Cm3701	Cm3701	Cm3701	Cm3701	Cm2200	Cm3701
**28S**	3715	Ψ3715	Ψ3715	Ψ3715	Ψ3715	Ψ2214	Ψ3715
**28S**	3718	Am3718	Am3718	Am3718	Am3718		Am3718
**28S**	3723	ND	Am3723	Am3723	Am3723	Am2223	Am3723
**28S**	3724			Am3724			
**28S**	3729	ND	Ψ3729	Ψ3729	Ψ3729		Ψ3729
**28S**	3734	ND	Ψ3734	Ψ3734	ND		
**28S**	3744	Gm3744	Gm3744	Gm3744	Gm3744		
**28S**	3758	ND	Ψ3758	Ψ3758	ND	Ψ2257	
**28S**	3760	ND	Am3760	Am3760	ND	Am2259	
**28S**	3762	ND	Ψ3762	Ψ3762	ND	Ψ2261	m^{1}Ψ3762

**28S**	3764	ND	Ψ3764	Ψ3764	ND	Ψ2263	Ψ3764
**28S**	3768	ND	Ψ3768	Ψ3768	ND	Ψ2267	
**28S**	3770	ND	Ψ3770	Ψ3770	ND	Ψ2269	
**28S**	3782	m5C3782	m5C3782	m5C3782	m5C3782	m5C2281	m^{5}C3782
**28S**	3785	Am3785	Am3785	Am3785	Am3785	Am2284	Am3785
**28S**	3792	Gm3792	Gm3792	Gm3792	Gm3792	Gm2291	Gm3792
**28S**	3808	Cm3808	Cm3808	Cm3808	Cm3808		
**28S**	3818	Ψm3818	Ψm3818	Ψm3818	Ψm3818	Ψ2317	
**28S**	3822	Ψ3822	Ψ3822		ND	Ψ2321	
**28S**	3825	Am3825	Am3825	Am3825	Am3825	Am2324	Am3825
**28S**	3830	Am3830	Am3830	Am3830	Am3830	Am2329	
**28S**	3841	Cm3841	Cm3841	Cm3841	Cm3841	Cm2340	
**28S**	3844	Ψ3844	Ψ3844	Ψ3844	Ψ3844		
**28S**	3851	Ψ3851	Ψ3851	Ψ3851	Ψ3851	Um2350	
**28S**	3853		Ψ3853	Ψ3853	Ψ3853		
**28S**	3867	Am3867	Am3867	Am3867	Am3867		Am3867
**28S**	3869	Cm3869	Cm3869	Cm3869	Cm3869	Cm2368	Cm3869
**28S**	3880						xp^{7}G3880
**28S**	3884	Ψ3884	Ψ3884	Ψ3884	Ψ3884		
**28S**	3887	Cm3887	Cm3887	Cm3887	Cm3887		Cm3887
**28S**	3897						ac^{7}G3897
**28S**	3899	Gm3899	Gm3899	Gm3899	Gm3899	Gm2398	ac^{7}Gm3899
**28S**	3909						Cm3909
**28S**	3920	Ψ3920	Ψ3920	Ψ3920	Ψ3920	Ψ2419	
**28S**	3925	Um3925	Um3925	Um3925	Um3925	Um2424	
**28S**	3944	ND	Gm3944	Gm3944	ND		
**28S**	3959	ND	Ψ3959	ND	ND		
**28S**	4042	ND	Gm4042	ND	ND		
**28S**	4054	ND	Cm4054	ND	ND		
**28S**	4083						m^{5}U4083
**28S**	4129						m^{6}G4129
**28S**	4185						m^{6}G4185
**28S**	4194						xp^{4}U4194
**28S**	4196	Gm4196	Gm4196	Gm4196	Gm4196	Gm2622	Gm4196
**28S**	4220	m6A4220	m6A4220	m6A4220	m6A4220		m^{6}A4220
**28S**	4227	Um4227	Um4227	Um4227	Um4227	Ψm2653	
**28S**	4228	Gm4228	Gm4228	Gm4228	Gm4228	Gm2654	
**28S**	4293	Ψ4293	Ψ4293	Ψ4293	Ψ4293		Ψ4293
**28S**	4296	Ψ4296	Ψ4296	Ψ4296	Ψ4296		m^{1}Ψ4296
**28S**	4299	Ψ4299	Ψ4299	Ψ4299	Ψ4299		
**28S**	4306	Um4306	Um4306	Um4306	Um4306	Um2732	Um4306
**28S**	4312	Ψ4312	Ψ4312	Ψ4312	Ψ4312	Um2738	
**28S**	4335						m^{5}C4335
**28S**	4353	Ψ4353	Ψ4353	Ψ4353	Ψ4353		

**28S**	4355						xe^{6}G4355
**28S**	4361	Ψ4361	Ψ4361	Ψ4361	Ψ4361		
**28S**	4370	Gm4370	Gm4370	Gm4370	Gm4370	Gm2796	Gm4370
**28S**	4371						m^{2}xp^{7}G4371
**28S**	4392	Gm4392	Gm4392	Gm4392	Gm4392	Gm2818	
**28S**	4403	Ψ4403	Ψ4403	Ψ4403	Ψ4403	Ψ2829	Ψ4403
**28S**	4415						m^{1}A4415
**28S**	4420	Ψ4420	Ψ4420	Ψ4420	Ψ4420		
**28S**	4423	Ψ4423	Ψ4423	Ψ4423	Ψ4423		
**28S**	4431	Ψ4431	Ψ4431	Ψ4431	Ψ4431	Ψ2857	
**28S**	4442	Ψ4442	Ψ4442	Ψ4442	Ψ4442	Ψ2868	Ψ4442
**28S**	4447	m5C4447	m5C4447	m5C4447	m5C4447	m5C2873	m^{5}C4447
**28S**	4450						Ψ4450
**28S**	4456	Cm4456	Cm4456	Cm4456	Cm4456	Cm2882	
**28S**	4457	Ψ4457	Ψ4457	Ψ4457	Ψ4457	Ψ2883	
**28S**	4471	Ψ4471	Ψ4471	Ψ4471	Ψ4471	Ψ2897	
**28S**	4472						m^{6}G4472
**28S**	4483						m^{4}C4483
**28S**	4493	Ψ4493	Ψ4493	Ψ4493	Ψ4493		
**28S**	4494	Gm4494	Gm4494	Gm4494	Gm4494	Gm2920	Gm4494
**28S**	4498	Um4498	Um4498	Um4498	Um4498	Um2924	
**28S**	4499	Gm4499	Gm4499	Gm4499	Gm4499	Gm2925	
**28S**	4500	Ψ4500	Ψ4500	Ψ4500	Ψ4500	Ψ2926	Ψ4500
**28S**	4521	Ψ4521	Ψ4521	Ψ4521	Ψ4521	Ψ2947	
**28S**	4523	Am4523	Am4523	Am4523	Am4523	Am2949	Am4523
**28S**	4529						m^{6}G4529
**28S**	4530	m3U4530	m3U4530	m3U4530	m3U4530	m3U2956	m^{3}U4530
**28S**	4531			Ψ4531			Ψ4531
**28S**	4532	Ψ4532	Ψ4532	Ψ4532	Ψ4532	Ψ2958	
**28S**	4536	Cm4536	Cm4536	Cm4536	Cm4536	Cm2962	Cm4536
**28S**	4550						m^{7}G4550
**28S**	4552	Ψ4552	Ψ4552	Ψ4552	Ψ4552	Ψ2978	
**28S**	4564						m^{7}A4564
**28S**	4569			Ψ4569			
**28S**	4571	Am4571	Am4571	Am4571	Am4571		Am4571
**28S**	4576	Ψ4576	Ψ4576	Ψ4576	Ψ4576		
**28S**	4579	Ψ4579	Ψ4579	Ψ4579	Ψ4579		
**28S**	4590		Am4590	Am4590	Am4590		
**28S**	4597						m^{3}U4597
**28S**	4618	Gm4618	Gm4618	Gm4618	Gm4618		
**28S**	4620	Um4620	Um4620	Um4620	Um4620		Um4620
**28S**	4623	Gm4623	Gm4623	Gm4623	Gm4623		Gm4623
**28S**	4628	Ψ4628	Ψ4628	Ψ4628	Ψ4628		Ψ4628
**28S**	4636		Ψ4636	Ψ4636	ND		Ψ4636

**28S**	4637	Gm4637	Gm4637	Gm4637	Gm4637		Gm4637
**28S**	4671						m^4C4671
**28S**	4673	Ψ4673	Ψ4673	Ψ4673	Ψ4673		
**28S**	4689	Ψ4689	Ψ4689	Ψ4689	Ψ4689		
**28S**	4690						ac^7G4690
**28S**	4870						Gm4870
**28S**	4872						m^2G4872
**28S**	4972	Ψ4972	Ψ4972	Ψ4972	Ψ4972		
**28S**	5001	Ψ5001	Ψ5001	Ψ5001	Ψ5001		
**28S**	5010		Ψ5010	Ψ5010	Ψ5010		
**5.8S**	14		Um14	Um14			Um14
**5.8S**	55	Ψ55	Ψ55	Ψ55	Ψ55		
**5.8S**	69	Ψ69	Ψ69	Ψ69	Ψ69		
**5.8S**	75	Gm75	Gm75	Gm75	Gm75	Gm78	
**uL2**	His-216	ox-His			ox-His		
**uL3**	His-245	met-His			met-His	met-His	
**uL15**	His-39	ox-His			ox-His		
**eL29**	Lys-5				met-Lys		
**eL40**	Lys-98	met-Lys			trimet-Lys	met-Lys	met-Lys
**eL42**	Lys-53				met-Lys		

Supplementary Table 6. Identified pseudouridines and their N3-binding partners

Position*	binding partner*	distance (Å)
**224**	water	2.92
**1536**	water	2.83
**1677**	phosphate 1676	2.64
**1683**	water	2.83
**1744**	water	2.78
**1779**	water	3.01
**1781**	water	2.62
**1782**	water	2.92
**1792**	water	2.89
**1860**	water	2.59
**1862**	water	2.75
**2508**	phosphate 2507	2.69
**2839**	water	3.07
**3637**	water	2.7
**3639**	water	2.8
**3695**	water	2.64
**3715**	U3713	2.85
**3818**	phosphate	2.87
**3822**	water	3.07
**3844**	water	2.77
**3851**	water	2.92
**3884**	water	3.2
**3920**	water	2.69
**4293**	ribose 4329	2.83
**4296**	water	2.75
**4299**	water	2.93
**4312**	water	2.63
**4353**	water	2.83
**4361**	water	2.59
**4403**	water	2.59
**4420**	water	2.51
**4423**	water	2.68
**4431**	water	2.93
**4442**	water	2.86
**4457**	water	2.72
**4471**	water	2.76
**4493**	ribose 4512	3.06
**4500**	water	2.68
**4521**	water	2.98
**4532**	water	2.91
**4552**	water	2.64
**4576**	water	3.09
**4579**	water	2.65

**4628**	water	2.66
**4673**	water	2.79
**4689**	water	3.05
**4972**	water	2.79
**5001**	water	2.71
**5S-55**	water	2.76
**5S-69**	water	2.8

*if not otherwise indicated, position numbers refer to a nucleotide in 28S rRNA

Supplementary Table 7: Comparison of identified polyamines in high-resolution cryo-EM structures

**Neighbouring nucleotide in human 28S rRNA**	**This work**	**Faille *et al.*** (10)	**Holm et al.** (11)	**Smirnova *et al.*** (12)
**Organism and cell line**	Human HeLa	Human HEK Expi 293F	Human HEK Expi 293F	Tobacco plant
**U1649**	PUT	H2O	SPD	H2O
**G4175**	PUT	Mg+H2O	Mg+H2O	H2O
**5.8S U80**	PUT	K+H2O	K+H2O	H2O
**G1680**	PUT	Mg+H2O	Mg+H2O	H2O
**G1532**	PUT	K+H2O	Mg+H2O	H2O
**G3875**	PUT	H2O	H2O	H2O
**G3918**	PUT	K+H2O	Mg+H2O	H2O
**C3882**	H2O	H2O	PUT	H2O
**A4203**	-	H2O	PUT	H2O
**A4192**	H2O	H2O	PUT	H2O
**G4400**	H2O	H2O	PUT	H2O
**U2070**	H2O	H2O	PUT	H2O
**C1306**	SPD	H2O	SPD	H2O
**U291**	SPD	H2O	Mg+H2O	A47
**A39**	SPD	Mg+H2O	SPD	SPD
**A328**	SPD	H2O	SPD	H2O
**C1666**	SPD	K+H2O	PUT	H2O
**G2808**	SPD	H2O	SPD	Mg+H2O
**U3884**	SPD	H2O	Mg+H2O	H2O
**G4425**	H2O	H2O	SPD	H2O
**G2862**	H2O	H2O	SPD	H2O
**U1470**	H2O	H2O	SPD	H2O
**G2297**	H2O	H2O	SPD	H2O
**U13**	-	H2O	SPD	H2O
**U432**	H2O	H2O	SPD	H2O
**U1528**	SPM	SPM	SPD	water*
**A47**	SPM	Mg + H20	Mg + H20	SPM
**A1736**	SPM	Mg+H2O	PUT- shifted	water
**G1586**	SPM	Mg+H2O	PUT	water
**C1607**	SPM	Mg+H2O	SPD	water
	*eL*29 Arg 14	*eL*29 Arg 14	*eL*29 Arg 14	SPM

*Inspection of the density suggests the presence of a polyamine instead of water (see Suppl. Figure 9D)

References

1. Punjani,A., Rubinstein,J.L., Fleet,D.J. and Brubaker,M.A. (2017) CryoSPARC: Algorithms for rapid unsupervised cryo-EM structure determination. *Nat Methods*, **14**, 290–296.
2. Afonine,P. V., Grosse-Kunstleve,R.W., Echols,N., Headd,J.J., Moriarty,N.W., Mustyakimov,M., Terwilliger,T.C., Urzhumtsev,A., Zwart,P.H. and Adams,P.D. (2012) Towards automated crystallographic structure refinement with *phenix.refine*. *Acta Crystallogr D Biol Crystallogr*, **68**, 352–367.
3. Tegunov,D. and Cramer,P. (2019) Real-time cryo-electron microscopy data preprocessing with Warp. *Nature Methods 2019 16:11*, **16**, 1146–1152.
4. Kimanius,D., Dong,L., Sharov,G., Nakane,T. and Scheres,S.H.W. (2021) New tools for automated cryo-EM single-particle analysis in RELION-4.0. *Biochem J*, **478**, 4169–4185.
5. Ermel,U.H., Arghittu,S.M. and Frangakis,A.S. (2022) ArtiaX: An electron tomography toolbox for the interactive handling of sub-tomograms in UCSF ChimeraX. *Protein Sci*, **31**, e4472.
6. Pettersen,E.F., Goddard,T.D., Huang,C.C., Meng,E.C., Couch,G.S., Croll,T.I., Morris,J.H. and Ferrin,T.E. (2021) UCSF ChimeraX: Structure visualization for researchers, educators, and developers. *Protein Science*, **30**, 70–82.
7. Zhang,K. (2016) Gctf: Real-time CTF determination and correction. *J Struct Biol*, **193**, 1–12.
8. Zhang,K. (2020) Gautomatch.
9. Scheres,S.H.W. (2012) RELION: Implementation of a Bayesian approach to cryo-EM structure determination. *J Struct Biol*, **180**, 519–530.
10. Faille,A., Warren,A.J. and Dent,K.C. (2023) The chemical landscape of the human ribosome at 1.67 Å resolution. biorXiv doi: https://doi.org/10.1101/2023.02.28.530191, 28 February 2023, pre-print: not peer-reviewed.
11. Holm,M., Natchiar,S.K., Rundlet,E.J., Myasnikov,A.G., Watson,Z.L., Altman,R.B., Wang,H.Y., Taunton,J. and Blanchard,S.C. (2023) mRNA decoding in human is kinetically and structurally distinct from bacteria. *Nature*, **617**, 200–207.
12. Smirnova,J., Loerke,J., Kleinau,G., Schmidt,A., Bürger,J., Meyer,E.H., Mielke,T., Scheerer,P., Bock,R., Spahn,C.M.T., *et al.* (2023) Structure of the actively translating plant 80S ribosome at 2.2 Å resolution. *Nat Plants*, **9**, 987–1000.
13. Natchiar,S.K., Myasnikov,A.G., Kratzat,H., Hazemann,I. and Klaholz,B.P. (2017) Visualization of chemical modifications in the human 80S ribosome structure. *Nature*, **551**, 472–477.
14. Taoka,M., Nobe,Y., Yamaki,Y., Sato,K., Ishikawa,H., Izumikawa,K., Yamauchi,Y., Hirota,K., Nakayama,H., Takahashi,N., *et al.* (2018) Landscape of the complete RNA chemical modifications in the human 80S ribosome. *Nucleic Acids Res*, **46**, 9289–9298.
